# miR408-5p and miR408-3p cooperatively reduce cadmium uptake and accumulation in rice

**DOI:** 10.1371/journal.pbio.3003811

**Published:** 2026-06-02

**Authors:** Fuxi Rong, Yaqi Zhang, Fangrui Ni, Lantian Zhang, Mingxin Yu, Zheyuan Hong, Muhammad Fahad, Yuxin Shen, Chuanjia Liu, Shengke Tian, Dezhi Wu, Liang Wu

**Affiliations:** 1 State Key Laboratory of Rice Biology and Breeding, Zhejiang Key Laboratory of Crop Germplasm Innovation and Utilization, College of Agriculture and Biotechnology, Zhejiang University, Hangzhou, Zhejiang, China; 2 Hainan Yazhou Bay Seed Laboratory, Hainan Institute, Zhejiang University, Sanya, Hainan, China; 3 MOE Key Laboratory of Environmental Remediation and Ecological Health, College of Environmental and Resource Sciences, Zhejiang University, Hangzhou, Zhejiang, China; 4 Yuelushan Laboratory, College of Agronomy, Hunan Agricultural University, Changsha, Hunan, China; 5 Zhongyuan Institute, Zhejiang University, Zhengzhou, Henan, China; The Sainsbury Laboratory, UNITED KINGDOM OF GREAT BRITAIN AND NORTHERN IRELAND

## Abstract

In plants, a subset of miRNA precursors can yield multiple mature miRNAs; however, how they simultaneously regulate a single biological process remains poorly understood. Cadmium (Cd) is a non-essential heavy metal toxic to plants, posing serious risks to human health via the food chain. As rice is a major dietary source of Cd, elucidating the molecular mechanisms underlying Cd accumulation is crucial for ensuring food safety. Here, we show that a pair of miRNAs derived from the *MIR408* precursor cooperatively represses Cd uptake in roots by targeting distinct genes, consequently reducing Cd accumulation in rice grains. miR408-5p inhibits translation of *Heavy metal-associated Isoprenylated Plant Protein 9* (*HIPP19*), which is specifically expressed in exodermis and endodermis cells and facilitates Cd binding. Meanwhile, miR408-3p cleaves *Uclacyanin 7* (*UCL7*) mRNA, leading to enhance the activity of superoxide dismutases (SODs), and increase production of reactive oxygen species (ROS), particularly hydrogen peroxide (H_2_O_2_), which in turn suppresses Cd absorption and accumulation. Furthermore, knockout mutants of *HIPP19* and *UCL7*, as well as *MIR408* overexpressing lines, exhibit significantly decreased Cd content in grains, while the accumulation of other essential metals remains comparable to that of wild-type plants. These findings establish a promising strategy for producing “low-Cd rice” without compromising agronomic traits for food safety.

## Abstract 2

Although subset of precursors can yield multiple mature miRNAs, how they simultaneously regulate a single biological process remains poorly understood. This study shows that dual mature miRNAs from one precursor regulate cadmium absorption and accumulation, with implications for producing low-cadmium rice.

## Introduction

In plants, endogenous gene expression is post-transcriptionally regulated by a class of small RNAs, namely microRNA (miRNA). miRNA gene is transcribed by RNA polymerase II, generating a single-stranded transcript that harbors a stem-loop precursor formed by base pairing between self-complementary foldback regions [1]. Mature miRNA residing in one of the arms of the miRNA precursor regulates targets via mRNA digestion or translation inhibition after entering an effector complex, whereas the opposing fragment of the precursor, called miRNA*, is usually inactive and degraded [2]. While most miRNA precursors give rise to one miRNA and corresponding miRNA*, mounting evidence shows that they may encode additional mature miRNA species at either the same or different arms [1,3–6]. Thus far, the role and function mechanisms of multiple miRNA products originating from a single hairpin structure remain elusive [3,6,7].

The rapid industrialization has led to an increase in heavy metal pollution in many countries, posing a severe threat to human health [8]. Among heavy metals, cadmium (Cd) is highly toxic to living organisms, even in trace amounts. Rice grains constitute a crucial source of Cd because rice is a staple food for Asian people and readily absorbs Cd from contaminated soil [8,9].

Specific transporter proteins for Cd uptake by rice have not yet been identified. Instead, Cd uptake and transfer primarily occur through essential metal transporters, notably involving natural resistance-associated macrophage protein (NRAMP), Zn-regulated transporter, and iron-regulated transporter-like proteins (ZRT-IRT-related protein, ZIP) family transporters [8]. Given that many characterized Cd transporters are not specific and may interfere with manganese (Mn), zinc (Zn), or iron (Fe) uptake or translocation, developing low Cd-accumulating rice cultivars without compromising crop fitness and yields poses a significant challenge [10].

Plants mitigate Cd-induced toxicity through metal chelation, a mechanism involving the biosynthesis of metal ligands known as phytochelates (PCs) [11]. PCs form small molecule complexes with Cd, potentially trapping Cd in roots and influencing subsequent Cd uptake or transport [12]. Recently, metallochaperones have been implicated in binding metal ions and transferring them to intracellular compartments, thereby modulating Cd detoxification in plants [13,14]. Heavy metal-associated isoprenylated plant protein (HIPP) represents a class of metal-binding metallochaperones characterized by a heavy metal-associated domain (HMA) and a C-terminal isoprenylation motif [15,16]. These features have been predicted to be required for metal-binding activity and to be involved in interactions with other proteins; however, it remains unknown whether HIPPs specifically bind and chelate Cd or other essential metals to facilitate heavy-metal uptake and transportation [16].

Copper (Cu) is an essential transition metal in plants. Specific families of plant miRNAs are designated as Cu-miRNAs because their accumulation is influenced by the environmental concentrations of Cu [17]. These miRNAs play a role in suppressing transcripts that encode blue Cu proteins [18]. miR408 is among one of the most conserved and abundant Cu-miRNAs in land plants [19]. Recent studies have demonstrated that miR408 overexpression significantly enhances plant biomass and grain yields in rice by regulating *UCL8*, a member of the *uclacyanin* (*UCL*) gene family associated with small blue copper proteins [20]. The miR408-*UCL8* module is suggested to be linked with Cu allocation to the chloroplast, thereby influencing the key electron transporter in the light reactions of photosynthesis [20]. However, the mechanism by which miR408 integrates Cu delivery and environmental signals to form a regulatory network for promoting photosynthesis remains elusive [20–22]. In addition, since both Cu and Cd are heavy metals, miR408 has been observed to respond to Cd treatments via transcriptome analysis in several plants [23–26]; however, the role of miR408 in Cd uptake and accumulation has not been determined, and whether it can be manipulated for the development of low-Cd rice resources has not been evaluated.

Recently, we identified another miRNA, miR408-5p, derived from a different arm of miR408-3p in the *MIR408* precursor in rice [6]. We demonstrated that miR408-5p is involved in auxin signaling and mediates leaf inclination by regulating *AUXIN/INDOLE ACETIC ACID 30*, a critical repressor in the auxin pathway, by switching action modes in rice [6]. In this study, we characterized *HIPP19* as another target of miR408-5p, and demonstrated that miR408-5p suppresses HIPP19 translation, which may decrease Cd binding and accumulation in exodermis and endodermis cells. In addition, we revealed that miR408-3p mediates *UCL7* transcript digestion, thereby interfering with reactive oxygen species (ROS) forms and consequent Cd uptake. Importantly, we observed decreased Cd content in grains while rice maintained comparable levels of other essential metals in *hipp19* and *ucl7* mutants, as well as *MIR408*-overexpressing lines. Our study on the roles of dual mature miRNAs from one precursor in the regulation of Cd absorption and accumulation not only provides insights into the molecular functions of miRNAs in plants, but also offers novel strategy for producing “low-Cd rice” germplasm to enhance food safety.

## Results

### *MIR408* is induced by Cd and involved in Cd uptake in rice

**Fig 1:**
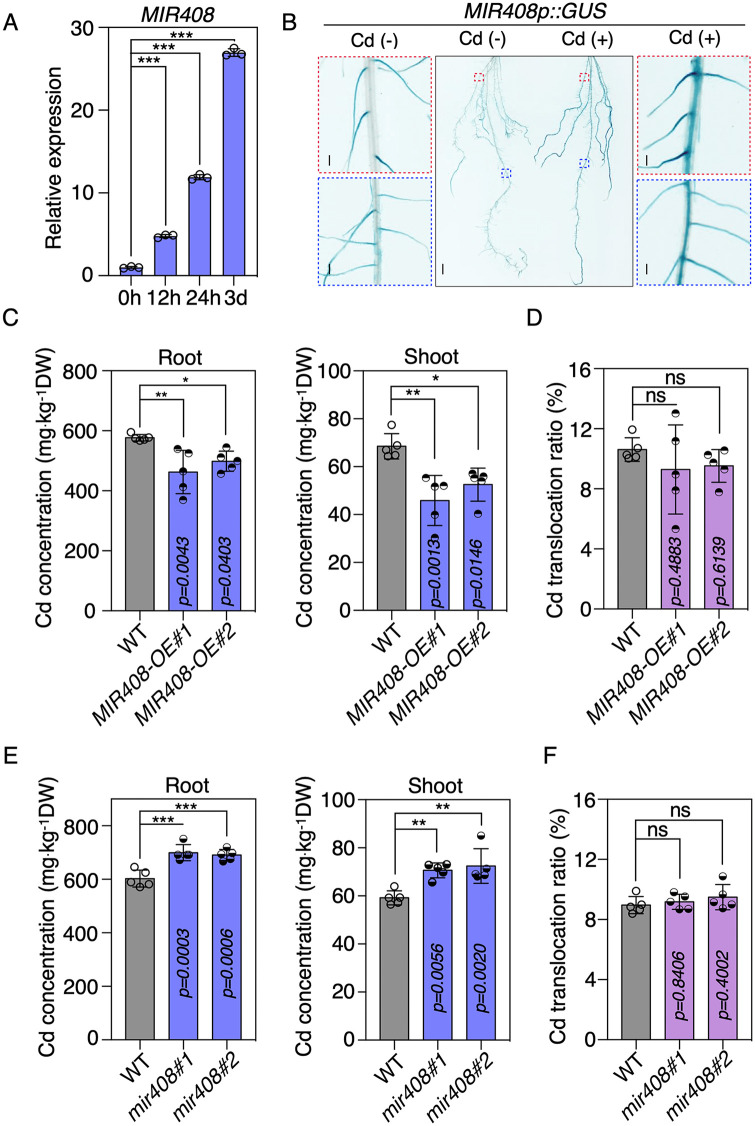
*MIR408* is involved in Cd uptake regulation in rice. **(A)** Time course analysis of pre-miR408 expressions in 14 d-old Nipponbare (Nip) plants with 2 μM CdCl_2_ treatment. *Actin* was used as an internal control for the normalization of the qRT-PCR results. Values are means ± SD (*n* = 3 biological replicates). **(B)** *MIR408p-GUS* staining in 10d-old rice plants with and without 4 h 2 μM CdCl_2_ treatment. Bars = 1 cm. **(C)** The contents of Cd in roots and shoots of 14 d-old WT and *MIR408-OE* seedlings grown in 2 μM CdCl_2_ conditions. **(D)** Translocation of Cd from roots to shoots of WT and *MIR408-OE* seedlings exposed in 2 μM CdCl_2_ for 14 days. ns, not signiﬁcant. **(E)** The contents of Cd in roots and shoots of 14 d-old WT and *mir408* mutant seedlings grown in 2 μM CdCl_2_ conditions. **(F)** Translocation of Cd from roots to shoots of WT and *mir408* mutants exposed in 2 μM CdCl_2_ for 14 days. Error bars in (C) to (F) indicate SD (Student’s *t* test, *P < 0.05; **P < 0.01; ***P < 0.001). (*n* = 5 biological replicates). The data underlying this Figure can be found in S1 Data.

To examine the response of *MIR408* to Cd, wild-type (WT) rice seedlings were first subjected to moderate Cd (2 μM) stress for 12–72 h. The expression of stem-loop miR408 precursor (pre-miR408) exhibited a significant increase, with the levels rising approximately 5-fold in roots after 12 h of treatment, while enhanced by over 25-fold after 3 days (Fig 1A). In a time-dependent experiment involving high-concentration Cd treatment (10 μM), an increased induction of pre-miR408 by Cd supply was observed (S1A Fig). In addition, GUS staining in both primary and adventitious roots from transgenic lines expressing the *GUS* reporter gene under the control of the *MIR408* promoter implicated a significant enhancement of primary miR408 (pri-miR408) transcription after 4h of moderate Cd treatment (Fig 1B). Taken together, these results suggest that the *MIR408* is strongly induced in Cd-exposed rice roots.

To investigate the potential involvement of *MIR408* in Cd regulation in rice, we measured Cd contents in roots and shoots of *MIR408-*overexpressing transgenic plants (*MIR408-OE*). In contrast with similar contents of Fe, Mn, Cu, and Zn (S1B and S1C Fig), the concentration of Cd in two-week-old transgenic plants was remarkably lower than that in WT plants after exposure to moderate Cd for 14 days, regardless of accumulation in roots or shoots (Fig 1C). Meanwhile, when we calculated the translocation ratio of Cd in shoots and roots, we found that it was comparable in WT and *MIR408-OE* plants (Fig 1D), indicating that ectopic expression of *MIR408* in rice suppresses the intake of Cd from roots, but may not affect the translocation efficiency of Cd from underground to aboveground.

To validate the involvement of *MIR408* in Cd absorption in rice, contents of Cd in roots and shoots were compared between WT and two independent *mir408* mutant lines. The concentration of Cd in roots increased from 600 to 700 mg kg^−1^ dry weight (DW), with a similar proportion of increase in shoots in *mir408* mutants compared with that in WT plants (Fig 1E and 1F), confirming the negative effects of *MIR408* on Cd uptake in rice. Moreover, we observed similar accumulations of Mn, Cu and Zn in roots and shoots, while only a slight decrease of Fe was observed in *mir408* roots compared with that in WT (S1D and S1E Fig), implying the feasibility of reducing Cd uptake and accumulation via *MIR408* manipulation in rice.

### miR408-5p regulates *HIPP19* via translation repression

**Fig 2:**
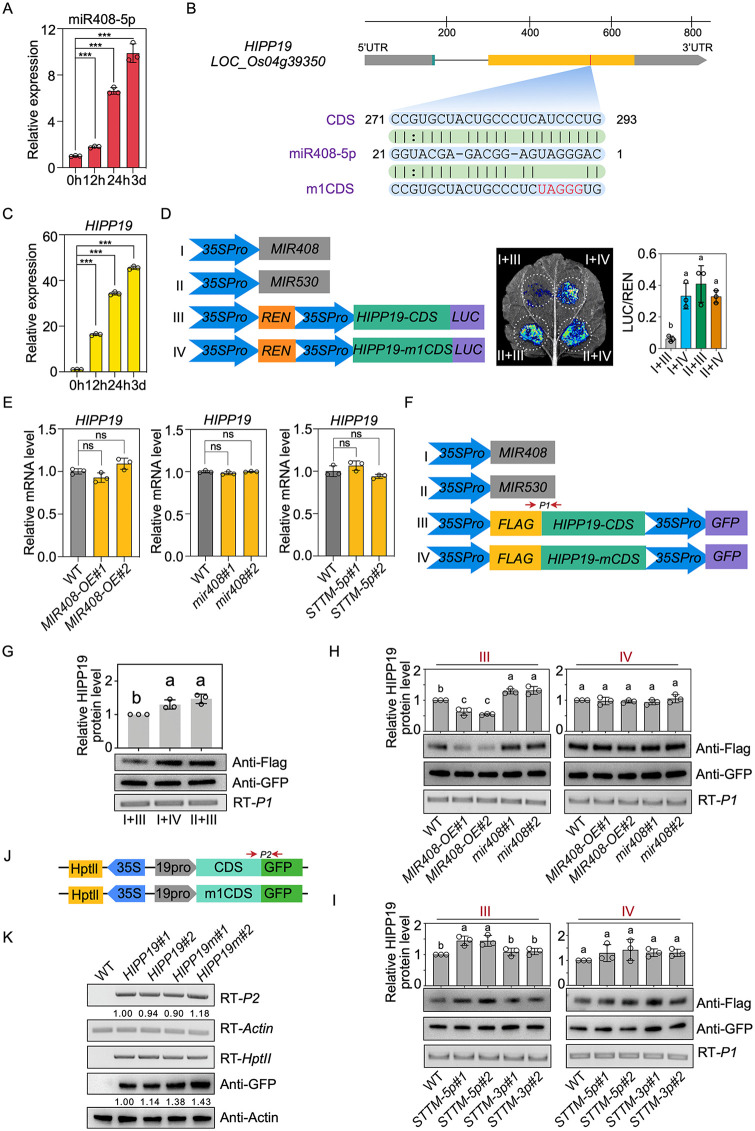
miR408-5p regulates *HIPP19* in a translation repression manner. **(A)** Time course analysis of miR408-5p accumulations in Nip plants with 2 μM CdCl_2_ treatment. Student’s *t* test, ***P < 0.001. Values are means ± SD (*n* = 3 biological replicates). **(B)** Gene structure of *HIPP19* and alignments of miR408-5p with target sites in *HIPP19* CDS and the indicated mutant CDS (m1CDS). **(C)** Relative mRNA levels of *HIPP19* in 14 d-old Nip plants with different time of 2 μM CdCl_2_ treatment. Values are means ± SD (*n* = 3 biological replicates). **(D)** Validation of *HIPP19* as miR408-5p target through transient expression analysis in *N. benthamiana* leaves. Left: The constructs in *A. tumefaciens* transiently introduced in *N. benthamiana* leaves. Middle: Representative photograph of ﬁreﬂy luciferase ﬂuorescence signals when the indicated construct combinations were introduced in *N. benthamiana* leaves. Right: Relative reporter activity in *N. benthamiana* leaves expressing the indicated construct combinations. Error bars indicate SD (Tukey’s honestly signiﬁcant difference, *P* < 0.05) (*n* = 3 biological replicates). **(E)** Relative mRNA levels of *HIPP19* in WT, *MIR408-OE*, *STTM-5p,* and *mir408* plants. ns, not signiﬁcant. Values are means ± SD (*n* = 3 biological replicates). **(F)** The constructs that were introduced into rice protoplast to determine the effect of miR408-5p on *HIPP19*. **(G)** The relative protein level of HIPP19 when *HIPP19* CDS or mCDS cassettes were introduced into rice protoplast with co-overexpression of *MIR408*. *MIR530* was used as a control. RT-P1 and immunoblot of GFP were used to show the efﬁciency of plasmid transiently introduced into the indicated protoplast. RT indicates transcript abundance determined by reverse transcription PCR. Anti denotes the protein level examined by western blot analysis using the indicated antibody (Tukey’s honestly signiﬁcant difference, *P* < 0.05). Values are means ± SD (*n* = 3 biological replicates). **(H)** The relative protein level of HIPP19 when *HIPP19* CDS or mCDS cassettes were introduced into rice protoplasts isolated from WT, *MIR408-OE,* and *mir408* plants. The different letters on top of each bar denote signiﬁcant differences (Tukey’s honestly signiﬁcant difference, P < 0.05). Values are means ± SD (*n* = 3 biological replicates). **(I)** The relative protein level of HIPP19 when *HIPP19* CDS or mCDS cassettes were introduced into rice protoplasts isolated from WT, *STTM-5p,* and *STTM-3p* plants. The experiments were performed three times and one of the representative results was shown below the columns (Tukey’s honestly signiﬁcant difference, *P* < 0.05). Values are means ± SD (*n* = 3 biological replicates). **(J)** The constructs were transformed into rice plants to determine the effect of miR408-5p on HIPP19. **(K)** RNA and protein abundance of *HIPP19* in WT and transgenic plants with indicated constructs shown in (J). The number below the lane represents the relative amounts of transcripts and proteins. The data underlying this Figure can be found in S1 Data and S1 Raw Images.

*MIR408* can produce two mature miRNAs, namely miR408-5p and miR408-3p, from different arms [6]. First, we determined the abundance of miR408-5p under moderate and high Cd treatments. Similar to the pri-miR408 and pre-miR408, we observed that miR408-5p was dramatically induced by environmental Cd in roots, particularly under the 3d long-term treatments (Figs 2A and S2A), implying a possible role of miR408-5p in Cd regulation in rice.

Using PsRobot, a widely-used small RNA analysis toolbox [27], we predicted a metallochaperone family gene, *HIPP19*, as a novel target of miR408-5p in rice (Figs 2B and S2B). Given that the expression of *HIPP19* similarly responds to Cd treatments (Figs 2C and S2C), we examined whether miR408-5p can regulate *HIPP19* in rice. To this end, we first performed a luciferase (LUC)-based reporter assay, wherein *HIPP19* was transiently expressed in *Nicotiana benthamiana* leaves under the control of 35S promoter with *MIR408* overexpression or with that of *MIR530* as a control (Figs 2D and S2D). We observed that the LUC signal generated by *HIPP19* activity was remarkably reduced when miR408-5p was overproduced compared with miR530 (Figs 2D and S2D). However, when we introduced mutated forms of *HIPP19* that were mismatched with miR408-5p target sites, the repressed LUC signal was restored (Figs 2D and S2D). These results suggested that *HIPP19* could be targeted and suppressed by miR408-5p.

Next, we investigated the expression of *HIPP19* in *MIR408-OE* transgenic plants and were surprised to observe that it was comparable to that in WT (Fig 2E). Furthermore, the *HIPP19* mRNA level exhibited similarity in both WT and *mir408* mutants (Fig 2E), in contrast with the significantly enhanced expression of *UCL8* (S2E Fig), a known miR408-3p target [20], when *MIR408* was knocked out. In addition, *HIPP19* exhibited a comparable expression pattern in WT and *Small Tandem Target Mimic-5p* (*STTM-5p*) transgenic plants with or without Cd treatment (Figs 2E and S2F), where miR408-5p accumulation was specifically repressed and miR408-5p activity was blocked (S2G Fig) [6]. This implies that miR408-5p has minimal effects on *HIPP19* transcription.

Since miR408-5p has been demonstrated to regulate targets via either mRNA cleavage or translation repression [6], we next determined whether miR408-5p inhibits HIPP19 translation. Initially, we co-expressed miR408 with intact or mutated *HIPP19* in rice protoplasts and examined the transcripts and proteins of *HIPP19* (Fig 2F). Although the GFP protein level was similar, which indicated comparable transformation efficiency, the abundance of HIPP19 protein shown by western blot using FLAG antibody was significantly lower when *MIR408* was co-expressed with intact *HIPP19* compared to that with a mutation at the target site or when *MIR408* was replaced to *MIR530* as a control (Fig 2G). Secondly, we transiently overexpressed *HIPP19* in the protoplasts of miR408 gain- and loss-of-function plants. As shown in Fig 2H, the protein level of HIPP19 was diminished in *MIR408-OE* and enhanced in *mir408* mutants, respectively. However, no altered HIPP19 protein level was observed when the mutated form of *HIPP19* was introduced instead of the intact version (Fig 2H). Considering that the mRNA level of *HIPP19* was similar in the protoplasts from all plants (Fig 2H), these data indicate that the downregulation of *HIPP19* by *MIR408* takes place at the protein level.

To demonstrate that *HIPP19* regulation is specific to miR408-5p, we introduced the plasmid described above into *STTM-5p* and *STTM-3p* plants, where the activity of miR408-5p and miR408-3p was respectively blocked [6]. Interestingly, the HIPP19 protein was significantly increased in the protoplasts from *STTM-5p* plants, while it remained consistent in *STTM-3p* plants compared with that in WT plants (Fig 2I). In addition, when the target site was mutated, the increased HIPP19 protein levels in *STTM-5p* disappeared (Fig 2I). In addition, when we introduced intact or mutated *HIPP19* at the miR408-5p target site with a GFP tag driven by the native *HIPP19* promoter (named *HIPP19* and *HIPP19m*, respectively) into rice (Fig 2J), we found that although the transcription of *HIPP19* was comparable (Fig 2K), its protein level was obviously higher in *HIPP19m* than in *HIPP19* transgenic plants (Fig 2K). A similar increase in HIPP19 protein accumulation in *HIPP19m* plants relative to *HIPP19* plants was observed under Cd treatment. Notably, although HIPP19 abundance was elevated in *HIPP19m* plants compared to *HIPP19* plants under Cd stress, this increase was less pronounced than that observed under normal growth conditions (S2H Fig). This discrepancy is likely attributable to the concurrent induction of both miR408-5p and *HIPP19* expression upon Cd exposure (Fig 2A and 2C)*.* These data collectively demonstrate that miR408-5p mediates *HIPP19* regulation through translation inhibition.

Since both miR408-5p and *HIPP19* are induced by Cd exposure (Fig 2A and 2C), we investigated whether Cd treatment alters the inhibitory effect of miR408-5p on HIPP19 protein accumulation. We isolated protoplasts from WT, *STTM-5p*, and *STTM-3p* plants with or without Cd exposure, then transiently overexpressed either intact *HIPP19* or *HIPP19* with mutated miR408-5p target sites in these protoplasts (Fig 2F). HIPP19 protein levels were significantly higher in *STTM-5p* protoplasts than in WT or *STTM-3p* protoplasts when overexpressing the intact *HIPP19*. However, Cd treatment attenuated this increase in *STTM-5p* plants compared to mock-treated controls (S2I Fig). Critically, when the miR408-5p target sites in *HIPP19* were mutated, HIPP19 protein levels became comparable across WT, *STTM-5p*, and *STTM-3p* protoplasts regardless of Cd exposure (S2I Fig). Given that miR408-5p is induced by Cd and its activity is blocked in *STTM-5p* plants, these results demonstrate that Cd exposure enhances miR408-5p-mediated repression of HIPP19 protein, dependent on functional target sites.

Collectively, these data demonstrate that miR408-5p suppresses HIPP19 via translational repression, and this inhibitory effect is potentially enhanced under Cd treatment.

### HIPP19 is involved in Cd uptake and accumulation regulation

**Fig 3:**
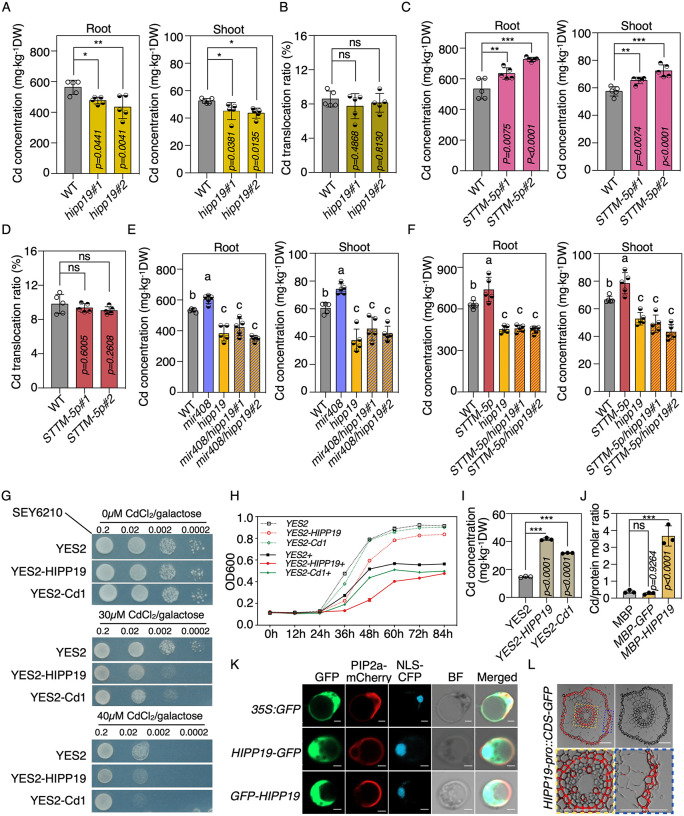
HIPP19 is important for Cd uptake and facilitates Cd accumulation in root exodermis and endodermis. **(A)** The contents of Cd in roots and shoots of 14 d-old WT and *hipp19* mutants grown in 2 μM CdCl_2_ conditions. Student’s *t* test, *P < 0.05; **P < 0.01. Values are means ± SD (*n* = 5 biological replicates). **(B)** Translocation of Cd from roots to shoots of WT and *hipp19* mutants exposed in 2 μM CdCl_2_ for 14 days. ns, not signiﬁcant. **(C)** The contents of Cd in roots and shoots of 14 d-old WT and *STTM-5p* plants grown in 2 μM CdCl_2_ conditions (Student’s *t* tes*t*, **P < 0.01; ***P < 0.001). **(D)** Translocation of Cd from roots to shoots of WT and *STTM-5p* plants exposed in 2 μM CdCl_2_ ns, not signiﬁcant. Values are means ± SD (*n* = 5 biological replicates). **(E)** The contents of Cd in roots and shoots of 14 d-old WT, *mir408*, *hipp19,* and *mir408*/*hipp19* mutants grown in 2 μM CdCl_2_ conditions (Tukey’s honestly signiﬁcant difference, *P* < 0.05). **(F)** The contents of Cd in roots and shoots of 14 d-old WT, *STTM-5p*, *hipp19,* and *STTM-5p*/*hipp19* plants grown in 2 μM CdCl_2_ conditions (Tukey’s honestly signiﬁcant difference, *P* < 0.05). **(G)** Dilution-series spot assays of yeast strain SEY6210 growth expressing *HIPP19*, *Cd1,* or empty vector YES2 in a medium containing different concentrations of Cd. **(H)** Growth of yeast strains shown in (G) with or without different time of 30 μM CdCl_2_ treatment. **(I)** Cd concentrations in yeast cells expressing *HIPP19*, *Cd1*, or empty vector YES2 after incubation in a liquid medium containing 30 μM Cd for 12 h (Student’s *t* test, ***P < 0.001). Values are means ± SD (*n* = 3 biological replicates). **(J)** In vitro Cd binding assay of HIPP19 to Cd. Full-leng*t*h HIPP19 recombinant proteins were extracted from BL21 and then incubated with 10 µM Cd for 4 h, pH = 7.4. MBP represents the *E. coli* trigger factor protein that fused to the N-terminus of the indicated proteins. GFP protein was used as a control (Student’s *t* test, ***P < 0.001). Values are means ± SD (*n* = 3 biological replicates). **(K)** Subcellular location analysis of HIPP19-GFP and GFP-HIPP19 in rice protoplasts. PIP2a-mCherry and NLS-CFP represen*t*s the localization in plasma membrane and nucleus, respectively. Bars = 5 μm. **(L)** Immunostaining of *HIPP19-GFP* transgenic rice roots under the control of *HIPP19* native promoter (*HIPP19pro::HIPP19CDS-GFP*). Immunostaining was performed using a GFP antibody. Red represents the signal. Bars = 100 μm. The data underlying this Figure can be found in S1 Data.

To investigate the physiological significance of the miR408-5p-*HIPP19* module in Cd regulation in rice, we generated *HIPP19* knock-out mutants using the CRISPR/Cas9 approach and measured Cd contents in roots and shoots of *hipp19* mutants grown in nutrient solution with 10-day 2 μM Cd supply (S3 Fig). Consistent with findings in *MIR408-OE* lines, we observed a remarkable decrease in Cd concentrations both in roots and shoots of *hipp19* mutants at the vegetative growth stage (Fig 3A), but the distribution of Cd between shoots and roots showed no obvious differences (Fig 3B). Additionally, 30 min and 2-hour short-term Cd exposure experiments showed decreased Cd contents in both roots and shoots of *hipp19* mutants compared to WT plants (S4 Fig), collectively demonstrating that HIPP19 mediates Cd uptake rather than long-distance translocation in rice.

In *mir408* mutants and *STTM-5p* plants, the Cd content was significantly elevated in both roots and shoots, and the distribution ratio was comparable to that of the WT (Fig 3C and 3D). However, when we knocked out *HIPP19* in *mir408* mutants or *STTM-5p* plants, their increased Cd accumulation decreased as that in *hipp19* mutants (Figs 3E, 3F, and S5A), suggesting that *HIPP19* is epistatic to *MIR408* and miR408-5p in Cd uptake regulation in rice.

To perform functional characterization of HIPP19, full-length *HIPP19* was cloned into the yeast expression vector pYES2 and transformed into the yeast SEY6210 strain [28]. Under Cd stress, the growth status of the HIPP19 strain was considerably worse than that of the control strain, similar to the strain with Cd1 introduction (Fig 3G and 3H), which has been identified as a facilitator for Cd uptake in rice [29]. In addition, the determination of Cd concentrations revealed that yeast strains expressing *HIPP19* accumulated much higher Cd than the control strains (Fig 3I), suggesting that heterologous expression of *HIPP19* suppressed Cd tolerance due to enhanced Cd uptake in yeast.

Given that HIPP19 contains a typical HMA domain, we investigated whether it can directly bind to Cd ions. In vitro metal binding assays revealed that while HIPP19 exhibits a capacity for Cd, which is more than 10-fold high than that of the control GFP proteins (Fig 3J). These results suggest that HIPP19 may interact with and form a stable complex with Cd in rice, similar to the observed association of phytochelatins with Cd and metal ions [30].

To determine the subcellular localization of HIPP19, we fused GFP to the N- or C-terminus of HIPP19 and transiently introduced them in rice protoplasts and *N. benthamiana* cells under the control of the *35S* promoter. Both GFP-HIPP19 and HIPP19-GFP were observed in the plasma membrane, cytoplasm, and nucleus (Figs 3K and S5B), implying that HIPP19 may transport or/and chelate Cd within the cells. To further confirm the cellular localization of HIPP19 in the root mature zone, we performed immunostaining using an antibody against GFP in *HIPP19* promoter-driven *HIPP19-GFP* transgenic rice plants [31,32]. As shown in Fig 3L, HIPP19 was notably observed in the outer regions of the exodermis and endodermis. Given that *hipp19* mutants exhibited reduced Cd accumulation in both shoots and roots (Fig 3A), it is possible that HIPP19 binds Cd and facilitate Cd accumulation in exodermis and endodermis cells, thereby augmenting Cd uptake in roots and accumulation in shoots in rice.

Collectively, our findings demonstrate that miR408-5p suppresses HIPP19 translation, potentially reducing HIPP19 accumulation in root exodermis and endodermis cells. This mechanism may impede Cd uptake in roots and consequently diminish Cd accumulation in shoots.

### miR408-3p modulates Cd uptake via targeting *UCL7*

**Fig 4:**
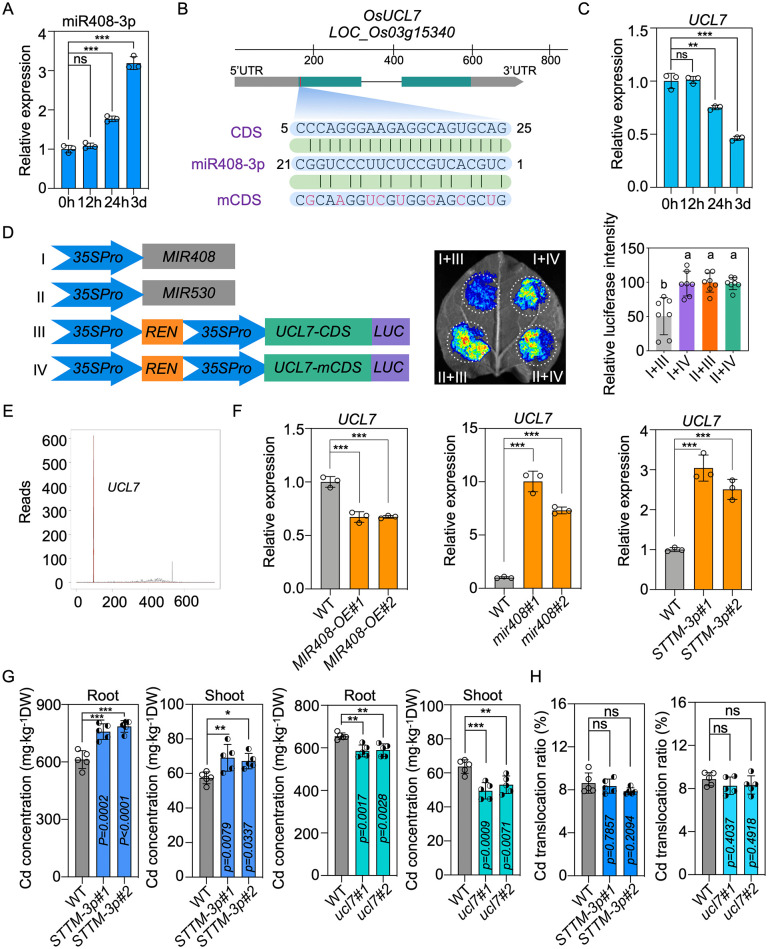
miR408-3p modulates Cd uptake via targeting *UCL7.* **(A)** Time course analysis of miR408-3p accumulations in Nip rice with 2 μM CdCl_2_ treatment. Student’s *t* test, *P < 0.05; **P < 0.01. Values are means ± SD (*n* = 3 biological replicates). **(B)** Gene structure of *UCL7* and alignments of miR408-3p with target sites in *UCL7* CDS and the indicated *UCL7* mutated CDS. **(C)** Time course analysis of *UCL7* expressions in Nip plants with 2 μM CdCl_2_ treatment. Values are means ± SD (*n* = 3 biological replicates). **(D)** Validation of *UCL7* as miR408-3p target through transient expression analysis in *N. benthamiana* leaves. Left: The constructs in *A. tumefaciens* transiently introduced in *N. benthamiana* leaves. Middle: Representative photograph of ﬁreﬂy luciferase ﬂuorescence signals when the indicated construct combinations were introduced in *N. benthamiana* leaves. Right: Relative reporter activity in *N. benthamiana* leaves expressing the indicated construct combinations. Error bars indicate SD (Tukey’s honestly signiﬁcant difference, *P* < 0.05). Values are means ± SD (*n* = 3 biological replicates). **(E)** Cleavage events of miR408-3p to *UCL7* mRNA shown by degradome sequencing data obtained from *MIR408-OE* transgenic plants. **(F)** Relative expressions of *UCL7* in WT, *MIR408-OE*, *mir408,* and *STTM-3p* plants. Values are means ± SD (*n* = 3 biological replicates). **(G)** The contents of Cd in roots and shoots of 14 d-old WT, *STTM-3p,* and *ucl7* plants grown in 2 μM CdCl_2_ conditions (Student’s *t* test, *P < 0.05; **P < 0.01; ***P < 0.001). Values are means ± SD (*n* = 5 biological replica*t*es). **(H)** Translocation of Cd from roots to shoots of WT, *STTM-3p,* and *ucl7* plants exposed in 2 μM CdCl_2._ ns, not signiﬁcant. Values are means ± SD (*n* = 5 biological replicates). The data underlying this Figure can be found in S1 Data.

In addition to miR408-5p, we observed the induction of miR408-3p by Cd treatment, although with a lower fold change compared to miR408-5p (Figs 4A and S6A). While miR408-3p has been documented to target a subset of *UCL* family genes in rice [20,33–35], our examination of potential *UCL* targets in response to Cd revealed that only *UCL7* was downregulated in rice roots following 2 μM Cd treatment (Figs 4B, 4C, and S6B). Given the inverse expressions of miR408-3p and *UCL7*, our subsequent investigation focused on the regulation of *UCL7* by miR408-3p involved in Cd accumulation.

Transient overexpression of *UCL7* alongside *MIR408* in *N. benthamiana* significantly suppressed the LUC signal derived from UCL7 protein, compared to co-expression with *MIR530* or a mutated *UCL7* construct (Fig 4B and 4D). This suggests that *UCL7* is repressed by mature miRNAs processed from pre-miR408. Searching for our previous degradome datasets [6], we revealed that the mRNA of *UCL7* undergoes cleavage by miR408-3p at a typical miRNA-target cleavage site (Fig 4E). Consequently, we assessed the expression of *UCL7* in miR408-3p gain- and loss-of-function plants. Compared to WT, *UCL7* transcripts were significantly decreased in *MIR408-OE* plants, whereas substantially enhanced in *mir408* mutants as well as in *STTM-3p* plants with or without Cd treatment (Figs 4F and S6C), where miR408-3p accumulation was specifically decreased and miR408-3p activity was blocked (S6D Fig) [6]. These findings strongly suggest that miR408-3p regulates *UCL7* in a mRNA digestion manner.

In line with the enhancement of Cd in *mir408* mutants, we found that the Cd levels in *STTM-3p* transgenic plants were increased, both in shoots and roots (Figs 4G and S6E). In contrast, Cd contents were detected decreased in *ucl7* mutants, regardless in roots or shoots (Figs 4G, S6F, and S6G). Nevertheless, the distribution ratio of Cd between shoots and roots remained identical in *STTM-3p* and *ucl7* plants, resembling the ratio observed in WT plants (Fig 4H). This supports the idea that miR408-3p is involved in Cd uptake rather than regulating Cd transportation through post-transcriptional control of *UCL7*.

### UCL7 regulates Cd intake and accumulation by affecting ROS metabolites

The similar regulatory roles of miR408-3p and miR408-5p in Cd uptake rather than in Cd translocation led us to investigate whether their respective targets, *UCL7* and *HIPP19*, share similar molecular contributions to Cd absorption in rice.

To achieve this, we first assessed the sensitivity of *UCL7*-transgenic yeast to Cd stress (S7A Fig). However, the findings indicated that the growth rate of yeast expressing *UCL7* remained unaltered in the Cd environment (S7A and S7B Fig). Furthermore, Cd accumulation in yeast expressing *UCL7* was similar to that in WT yeast, considerably lower than in yeast expressing Nramp 5 (S7C Fig), a recognized Cd transporter [36], suggesting that UCL7 does not function as a Cd transporter.

Next, we purified the UCL7 protein in *E.coli* and performed in vitro metal binding assays to examine its metal binding capabilities. Notably, interactions between UCL7 and Cu were readily detected (S7D Fig), aligning with the established understanding that UCLs are small copper protein [37]. By contrast, associations of UCL7 with Cd ions could not be detected (S7D Fig). These findings collectively imply that the molecular mechanism underlying the reduction of Cd content in *ucl7* roots and shoots differs from that in *hipp19* mutants.

**Fig 5:**
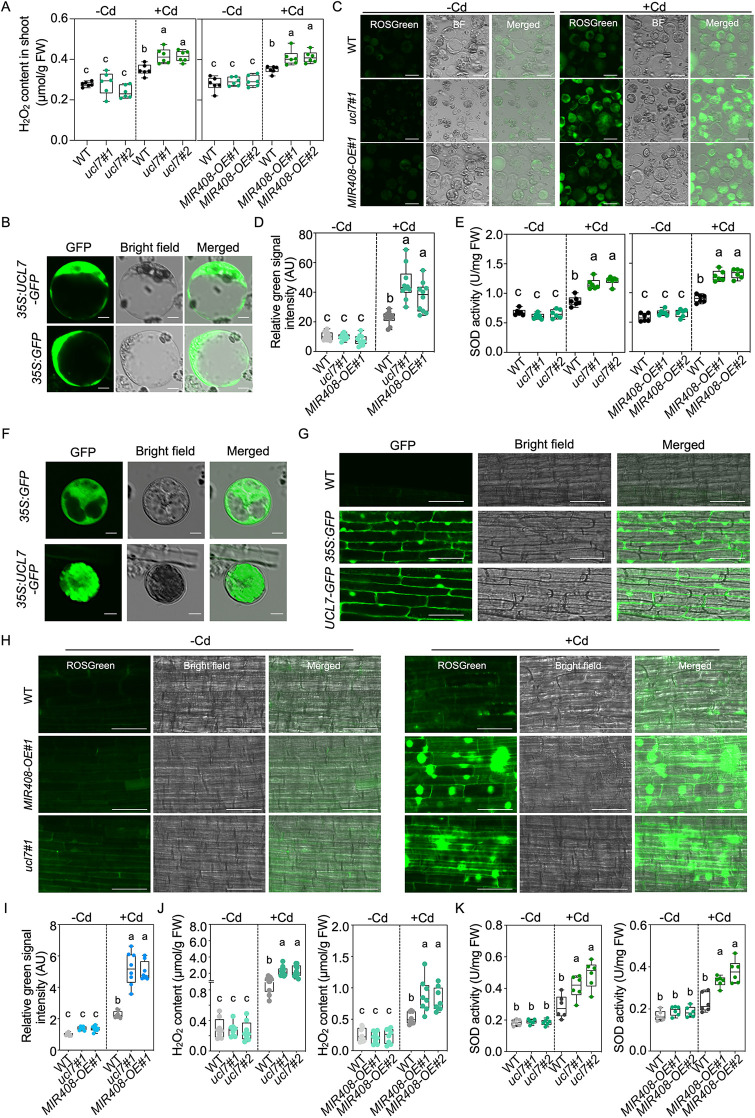
UCL7 regulates Cd intake by affecting SOD activity and H_2_O_2_ production. **(A)** Quantification of H_2_O_2_ contents in the shoot of WT, *ucl7*, and *MIR408-OE* seedlings exposed to mock and 2 μM CdCl_2_ conditions for 14 days (Tukey’s honestly signiﬁcant difference, *P* < 0.05). Values are means ± SD (n = 6 biological replicates). **(B)** Subcellular location of UCL7-GFP in protoplasts isolated from rice shoots. Bars = 5 μm. **(C)** Green fluorescent signal generated by ROSGreen dye in protoplasts isolated from WT, *MIR408-OE,* and *ucl7* seedlings with or without 2 μM CdCl_2_ treatment. Bars = 25 μm. **(D)** The relative intensity of the fluorescent signal shown in **(C)** (Tukey’s honestly signiﬁcant difference, *P* < 0.05). Values are means ± SD (*n* = 10 biological replicates). **(E)** Quantification of SOD activities in the shoots of WT, *ucl7*, and *MIR408-OE* seedlings exposed to mock and 2 μM CdCl_2_ conditions for 14 days (Tukey’s honestly signiﬁcant difference, *P* < 0.05). Values are means ± SD (*n* = 6 biological replicates). **(F)** Subcellular location of UCL7-GFP in protoplasts isolated from rice root tips. Bars = 5 μm. **(G)** Subcellular location of UCL7-GFP in rice root cells. Bars = 100 μm. **(H)** Green fluorescent signal generated by ROSGreen dye in root cells of WT, *MIR408-OE,* and *ucl7* seedlings with or without 2 μM CdCl_2_ treatment. Bars = 100 μm. **(I)** The relative intensity of the fluorescent signal shown in **(H)** (Tukey’s honestly signiﬁcant difference, *P* < 0.05). Values are means ± SD (*n* = 8 biological replicates). **(J)** Quantification of H_2_O_2_ contents in the roots of WT, *ucl7,* and *MIR408-OE* plants exposed to mock and 2 μM CdCl_2_ conditions for 14 days (Tukey’s honestly signiﬁcant difference, *P* < 0.05). Values are means ± SD (*n* = 8 biological replicates). **(K)** Quantification of SOD activities in the roots of WT, *ucl7,* and *MIR408-OE* roots exposed to mock and 2 μM CdCl_2_ conditions for 14 days (Tukey’s honestly signiﬁcant difference, *P* < 0.05). Values are means ± SD (n = 6 biological replicates). The data underlying this Figure can be found in S1 Data.

ROS signaling serves as a small-molecule secondary messengers in plant development and responses to environmental stresses [38]. Recent studies have highlighted the significance of the reactive oxygen species (ROS) signaling cascade in facilitating plant adaptation to toxic heavy metals in soil [39], including Cd and aluminum, therefore we sought to determine whether UCL7 regulates Cd uptake and accumulation via influencing ROS products. We assessed the hydrogen peroxide (H_2_O_2_) content, as H_2_O_2_ possesses the longest half-life among ROS [40]. As depicted in Fig 5A, although H_2_O_2_ accumulation was comparable without Cd treatment in WT and *ucl7* mutants, a significantly higher accumulation was observed in the shoots of *ucl7* compared to WT under Cd regime, suggesting that the regulation of ROS homeostasis in response to Cd sensing is abnormal when *UCL7* is deficient in rice. Additionally, under Cd stress, a similar enhancement of H_2_O_2_ accumulation was observed in *MIR408-OE* transgenic plants through H_2_O_2_ quantification (Fig 5A). This implies that the overproduction of miR408-3p may influence *UCL7* expression and control subsequent ROS signaling in rice in response to Cd toxicity.

UCL8, the closest ortholog of UCL7 in rice, has been demonstrated to subcellularly localize in the cytoplasm, modulating plastocyanin content and photosynthesis [20]. Similarly, the fluorescence signal in protoplasts isolated from rice shoots expressing UCL7-GFP indicates localization of UCL7 in the cytoplasm (Fig 5B). Considering that the cytoplasm is one of the locations that produce ROS [38,41,42], we assessed ROS accumulation in cell cytoplasm of rice shoots with or without Cd treatment. Fluorescent dyes, ROSGreen, have been used to visualize cellular H_2_O_2_ in plants [43,44]. Under Cd treatment, we noted a significant increase in green fluorescent signals (Fig 5C and 5D). This observation implies a substantial induction of H_2_O_2_ accumulation, representing a source of ROS, in response to Cd treatment in rice shoot cells. Strikingly, upon assessing the fluorescence intensity that reflected H_2_O_2_ levels in WT, *MIR408-OE* transgenic plants, and *ucl7* mutants after Cd treatment, we observed a pronounced increase in those plants overexpressing miR408-3p or harboring *ucl7* deletions (Fig 5C and 5D), indicating that UCL7 represses H_2_O_2_ generation in rice, especially under Cd conditions.

Superoxide dismutase (SODs) represent a family of metalloenzymes responsible for catalyzing the dismutation of superoxide radicals into H_2_O_2_ [38]. Given that the cytoplasm is one of primary sites for SODs distribution [45,46], we sought to investigate the potential impact of the miR408-3p-*UCL7* module on SOD enzyme activity in rice. Interestingly, we observed a significant increase in the activity of SOD in *MIR408-OE* plants compared to WT when subjected to Cd stress (Fig 5E). However, this heightened activity was not evident under normal water culture growth conditions (Fig 5E). Similarly, SOD activity in *ucl7* mutants demonstrated an increase compared with WT under Cd treatment, while remaining consistent in the absence of Cd exposure (Fig 5E). These findings imply that the induction of miR408-3p accumulation and the suppression of *UCL7* expression by Cd lead to enhance SOD activity in rice.

Because the overexpression of *MIR408* and deletion of *UCL7* block Cd uptake in rice roots (Figs 1C and 4G), we next attempted to determine the localization of UCL7 in root cells in addition to shoot cells. Even though isolation of protoplasts from roots was much more difficult than that from shoots, we successfully obtained transgenic protoplasts from root tips, and observed that UCL7-GFP was localized in cytoplasm of root cells as that in leaves (Fig 5F and 5G). Similar to shoot cells, the cellular H_2_O_2_ was limited to be accumulated in untreated roots, irrespective of WT, *MIR408-OE* and *ucl7* mutants, since fluorescent dyes of ROSGreen in them were difficult to be observed without Cd treatment (Fig 5H and 5I). In contrast, the green signal intensity of them was dramatically enhanced when rice plants were subject to 2 μM CdCl_2_ regimes (Fig 5H and 5I). Moreover, the measured H_2_O_2_ contents in *MIR408-OE* and *ucl7* roots displayed much higher than those in WT root cells (Fig 5J), and the cause of this observation was similar to that we found in shoots, because the measured SOD activity in roots was substantially increased in *MIR408-OE* transgenic plants and *ucl7* mutants compared to WT rice when plants were grown in Cd environments (Fig 5K).

Taken together, these data demonstrate that the inhibition of *UCL7* by miR408-3p enhances SOD enzyme activity and promotes H_2_O_2_ production, ultimately leading to impair Cd absorption and accumulation in rice.

### *MIR408* and targets enable the generation of low-Cd rice without yield penalty

**Fig 6:**
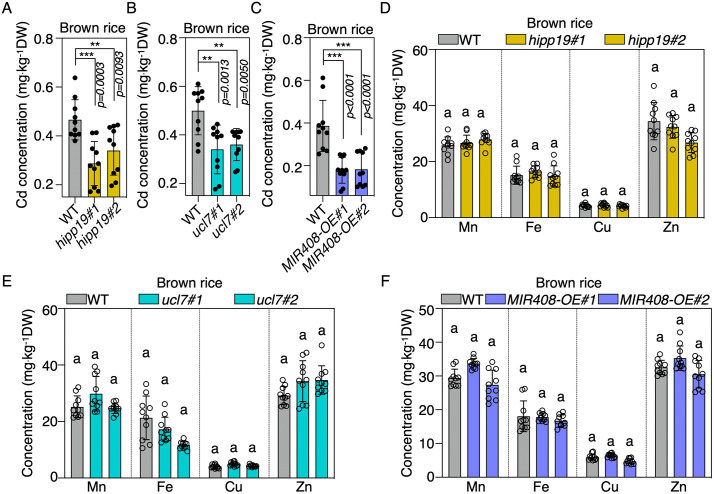
Manipulation of *MIR408* and targets enables the generation of low-Cd rice. **(A–C)** The concentration of Cd in brown rice of *hipp19* (A), *ucl7* (B), and *MIR408-OE* (C) plants compared to WT when grown in soil containing 2 mg kg^−1^ Cd (Student’s *t* test, **P < 0.01; ***P < 0.001). Values are means ± SD (*n* = 10 biological replicates). **(D–F)** The concentration of Mn, Fe, Cu, and Zn in brown rice of *hipp19* (D), *ucl7* (E), and *MIR408-OE* (F) plants compared to WT when they were grown in soil containing 2 mg kg^−1^ Cd (Tukey’s honestly signiﬁcant difference, P < 0.05). Values are means ± SD (*n* = 10 biological replicates). The data underlying this Figure can be found in S1 Data.

The primary source of Cd in rice grains is the soil. Given the significant decrease in Cd uptake in roots through the overexpression of *MIR408* and the deletion of *HIPP19* or *UCL7* in rice, we sought to investigate their impact on Cd accumulation in rice grains and their potential for generating low-Cd rice. To this end, experiments were conducted in large plastic pots with soil artificially contaminated with Cd (2 mg/kg), where both WT and transgenic or mutant rice were planted together. Inductively coupled plasma mass spectrometry (ICP-MS) determination of Cd contents revealed a significant decrease of Cd accumulation in brown rice in *hipp19* and *ucl7* mutants (Fig 6A and 6B). This indicates that the deficiency of *HIPP19* and *UCL7* interferes with Cd uptake, resulting in a block of Cd accumulation in grains. More interestingly, overexpression of *MIR408*, which leads to a simultaneous enhancement of miR408-5p and miR408-3p, accumulates much less Cd in brown rice than in *hipp19* and *ucl7* single mutants (Fig 6A–6C). This suggests that the dual effects of *MIR408* overproduction, inhibiting the protein level of HIPP19 and the transcription level of *UCL7* concurrently, can be explored more efficiently to create low-Cd germplasm to reduce grain Cd accumulation.

At the same time, the contents of essential minerals, including Mn, Fe, Cu, and Zn, were all comparable in the grains of *MIR408* overexpression lines, *hipp19* and *ucl7* mutants, compared with the WT, implying a significant potential for their application in the generating low-Cd rice (Fig 6D–6F). In addition, under natural field conditions, the plant architecture, and key agricultural traits, including plant height, 1,000-grain weight, number of effective panicles per plant, and number of grains per panicle, were similar between the *hipp19* and *ucl7* mutants and the WT plants (S8A–S8C Fig). Together with the previous finding that overexpression of *MIR408* remarkably enhances grain yield in rice and several other plants [20,21,47–49], the *MIR408* overexpression lines, *hipp19,* and *ucl7* mutants generated in this study enable the production of germplasm for low-Cd rice breeding without negative effects on grain yield and agronomical traits.

## Discussion

**Fig 7:**
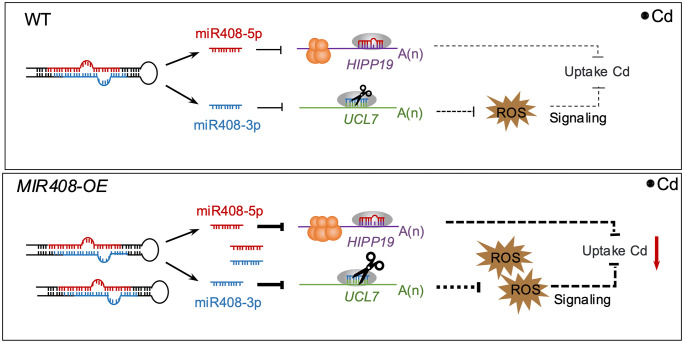
A working model of Cd uptake and accumulation regulation involving two mature miRNAs generated from *MIR408* in rice. The miR408 precursor generates two mature miRNAs: miR408-5p, which represses the translation of HIPP19, and miR408-3p, which cleaves UCL7 mRNA. Consequently, in transgenic plants overexpressing *MIR408*, elevated levels of miR408-5p suppress HIPP19 protein accumulation, while increased miR408-3p represses *UCL7* expression and promotes ROS accumulation. These effects act synergistically to reduce Cd uptake and accumulation compared with WT plants.

With the development of sequencing technology, increasing evidence has shown that more than one mature miRNAs can be generated from a single hairpin structure. However, the functions of these miRNAs, particularly their relevance in specific biological processes, remain largely elusive. Previously, the piece of evidence showed that miR393 (miR393-5p) and miR393* (miR393-3p) jointly regulate plant innate immunity by repressing auxin receptors and a Golgi/post-Golgi compartment-localized protein, respectively [50,51]. Here, we show that two mature miRNAs from the miR408 stem-loop regulate Cd uptake and accumulation by modulating different targets and through different molecular mechanisms (Fig 7). Together, these findings advance our understanding that eukaryotes may enhance regulatory efficiency for a biological event by transcribing and processing an RNA hairpin structure, which can subsequently produce multiple mature miRNAs that regulate different targets.

Although we have delineated the roles of miR408-5p and miR408-3p in Cd regulation, it is noteworthy here that rice likely lacks Cd-specific regulatory mechanisms. This is because rice evolved in environments historically is free of Cd contamination. Given that contemporary Cd pollution stems from recent anthropogenic activities, the *MIR408*-mediated regulatory response to Cd exposure observed in this study likely represents an adaptive mechanism originally evolved for other environmental cues, rather than a dedicated Cd detoxification strategy.

### The potential mechanism of HIPP19 in regulating Cd uptake and accumulation in rice

As a large family of heavy metal-associated proteins, HIPPs have been implicated in plant growth, development, and stress responses by delivering metals to transporters for metal distribution or detoxification [52–55]. A previous study showed that rice HIPP9 does not mediate Cd uptake during the vegetative growth stage, but mediates Cd translocation and restricts Cd accumulation in grains at the reproductive stage [16]. In contrast, our study reveals that HIPP19, which is targeted by miR408-5p, is expressed in root exodermis and endodermis cells, and plays a role in Cd binding and Cd uptake in rice. Strikingly, knockout of *HIPP19* lead to decreased, rather than increased, Cd accumulation in grains, suggesting that HIPP19 and HIPP9 play distinct roles in Cd regulation. Given that *HIPPs* constitute a large gene family in rice, and *HIPP9* and *HIPP19* belong to distinct subclades [53], we proposed that their distinct tissue expression patterns and amino acid compositions may determine their specialized functions in Cd uptake and translocation. Similarly, HMA3, a P_1B_-type ATPase, can also sequester Cd, but it detains Cd in the vacuole, which contributes to Cd translocation and accumulation in rice [28,56]. This suggests that subcellular compartmentalization and cell-type specificity endow metallochaperones with distinct roles in Cd uptake and transport.

To date, Nramp5 has been identified as the major transporter mediating Cd uptake in rice roots [36,57]. Our results showed that while the tissue localization of HIPP19 partially overlapped with that of Nramp5, both are expressed in the exodermis and endodermis [36] (Fig 3L), the two proteins did not physically interact (S9 Fig). We observed that HIPP19 enhances Cd absorption in yeast cells and localizes to the plasma membrane in addition to the cytoplasm (Figs 3K and S5B). These findings imply that HIPP19 may either directly mediate Cd uptake into cells or facilitate this process indirectly by interacting with other proteins.

Additionally, given that HIPP19 localizes to the cytoplasm and can directly bind Cd²⁺ (Fig 3J), we also propose that a negative feedback mechanism of HIPP19 regulation may be involved within the cell. In WT rice, HIPP19 may bind to Cd²⁺ to form a HIPP19-Cd²⁺ complex, thereby reducing and buffering the levels of free Cd²⁺ in the cytosol. This buffering effect likely mitigates immediate Cd toxicity and modulates Cd-related signaling. In contrast, in the *hipp19* mutant or miR408-5p gain-of-function transgenic plants, where HIPP19 activity is lost or attenuated, this Cd²⁺-binding capacity is impaired, possibly leading to elevated levels of unbound cytosolic Cd²^+^. We hypothesize that this increase in free Cd²⁺ may activate cytosolic heavy metal-sensing pathways, which in turn negatively regulate the expression or activity of metal influx transporters, ultimately reducing further Cd uptake.

Therefore, we propose two alternative mechanisms by which HIPP19 may modulate Cd uptake in rice (S10 Fig). First, the localization of HIPP19 to the plasma membrane likely facilitates Cd uptake into cells [58]. Second, cytoplasmic HIPP19 binds to Cd²⁺ and thus perhaps buffer the levels of free Cd²⁺ in the cytosol, which may ultimately trigger feedback regulation of heavy metal-sensing pathways. Further studies are required to elucidate the detailed mechanism by which HIPP19 mediates Cd uptake and accumulation in rice.

In addition, HIPP19 was also detected in xylem cells and the plasma membrane at the cellular and subcellular level (Fig 3L), suggesting that HIPP19 may also play roles in Cd xylem loading or xylem-to-phloem transfer, in addition to its involvement in Cd uptake and accumulation in root cells, although the distribution of Cd in roots and shoots of *hipp19* mutants was not observed altered compared to WT plants (Fig 3B). We speculate that HIPP19 functions in Cd transfer may overlap with other HIPP family proteins, particularly HIPP20, given their high protein similarities (S11 Fig).

While Cd treatment simultaneously induces the expression of both *HIPP19* and miR408-5p may appear counterintuitive, this regulatory configuration is biologically plausible. On one hand, *HIPP19* is transcriptionally upregulated in response to Cd exposure. On the other hand, the concurrent accumulation of miR408-5p acts post-transcriptionally to repress HIPP19 translation. These two opposing regulatory layers likely function in concert to maintain HIPP19 protein within an optimal range, which is critical for Cd stress response in rice. In the absence of miR408-5p-mediated repression, the transcriptional activation of *HIPP19* could become excessively pronounced, potentially leading to Cd hyperaccumulation and consequent detrimental effects on plant growth. Thus, while transcriptional induction of *HIPP19* is present, it does not diminish the importance of its post-transcriptional fine-tuning by miR408-5p. This balanced regulatory circuit ensures that HIPP19 is maintained at a level conducive to effective Cd stress management.

### The potential mechanism of UCL7 in regulating Cd uptake and accumulation in rice

UCL8, which is regulated by miR408-3p, has been shown localized to the plasma membrane, and affects photosynthesis and grain yield by regulating copper homeostasis [20]. Here, we found that *UCL7*, another target of miR408-3p, suppressed SOD activity, thereby decreasing H_2_O_2_ production in the cytoplasm. Cd induces *MIR408* transcription and miR408-3p accumulation, which enhances the cleavage of *UCL7* transcripts and thereby reduces the pool of *UCL7* transcripts available for translation. Given that UCL7 protein binds Cu²⁺ (S7D Fig), we conjecture this, in turn, may increase intracellular copper availability, consequently boosting Cu/Zn SOD activity. These sequential events may ultimately promote localized H_2_O_2_ production. Since H_2_O_2_ is widely recognized as a key physiological signal in plants [38,40,59], the enhanced H_2_O_2_ generated in roots and shoots is likely to affect cation channels or modulate heavy metal-related protein modifications, ultimately resulting in the inhibition of Cd uptake (S12 Fig) [60–62].

Nevertheless, the detailed molecular evidence regarding how H_2_O_2_ and ROS signal directly mediates Cd and heavy-metal absorption awaits further exploration. Given that MnSOD is known to accumulate under Cu deficiency conditions, whether MnSOD in addition to Cu/Zn SOD is involved in ROS accumulation activation under Cd treatment requires to be determined. Because many copper protein genes are predicted as targets of miR408-3p [63], it is possible that other UCLs or phytocyanin, besides UCL7, are regulated by miR408-3p and play roles in ROS manufacture and Cd uptake in rice roots. Additionally, we detected that miR398, which targets Cu/Zn SOD-encoding genes, was induced by Cd treatment (S13 Fig). Thus, whether miR408-3p and miR398 synergistically regulate Cu/Zn SOD activity to influence Cd uptake and accumulation warrants future investigation. Finally, as HIPP19 possesses heavy metal-binding activity and may bind Cu, we cannot exclude the possibility that it similarly modulates Cu/Zn SOD activity and ROS accumulation under Cd exposure.

### Development of low-Cd rice via harnessing *MIR408*-derived miRNAs and their targets

Over the past two decades, several genes involved in Cd uptake, translocation, and distribution have been characterized in rice; however, almost none of these genes have been directly utilized in breeding programs to date because they often play roles in the intake or transport of essential elements [36,56,64–69]. Therefore, simply knocking out these genes in rice often raises the dilemma of balancing between reducing Cd content and compromising yields due to the reduced accumulation of essential metals. For instance, Cd accumulation has been detected to be significantly suppressed in *nramp5* mutants, but plant growth was somehow destroyed due to reduced Mn intake, although a few variations of *NRAMP5* have been utilized in low-Cd rice generation [36,57]. Identifying genotypic variations and selecting superior alleles of genes involved in Cd accumulation may be an effective strategy for genetic improvement of rice; however, generating potential breeding materials for breeding rice varieties through marker-assisted backcrossing remains a time-consuming endeavor [29]. Therefore, another significant finding of our study is to provide an alternative approach to develop low-Cd rice. This includes knock-out mutants of *HIPP19* and *UCL7* and rice lines overexpressing *MIR408*, none of which show any alteration in essential metals or crop major traits (Fig 6). In particular, without a repulsive issue with transgenic plants, low-Cd rice varieties could be directly produced by knocking out *UCL7* and *HIPP19* in a variety of high-yield cultivars by gene-editing. Hence, we presume that our findings lay the groundwork for the development of crop varieties using gene-editing approaches to produce cultivars that are low in Cd content and without yield penalties, thereby addressing crucial global food security concerns.

## Materials and methods

### Plant material and growth conditions

The seeds of the Japonica rice (*Oryza sativa*) variety Nipponbare (Nip) were subjected to a surface sterilization process and soaked in a 3% sodium hypochlorite (NaClO) solution for 30 min. Afterward, the seeds were rinsed three to four times with sterilized deionized water (ddH_2_O). We then imbibed the seeds in distilled water for 48 h in dark at 37 °C to initiate germination. These germinated seeds were subsequently planted in a hydroponic box with 96 wells (manufactured by LabStar Company, Jiangsu), which were filled with Yoshida nutrient solution maintained at a pH of 5.8. After 4 days of growth, the seedlings were exposed to a 2 μM Cd solution at specified concentrations for 10 days. This exposure occurred in a controlled environment with a 14-h light (28 °C) and 10-h dark (22 °C) cycle. Alternatively, rice seedlings that reached the three-leaf stage were transplanted into pots filled with paddy soil containing 2 mg/kg of Cd. These plants were grown until the harvesting stage at the Agricultural Experiment Station of Zhejiang University.

### Construction and genetic materials

The coding sequences of *HIPP19* and *UCL7* were amplified from rice cDNA via polymerase chain reaction (PCR) and constructed into a modified pCAMBIA1390 vector under the regulation of a maize ubiquitin (*UBI*) promoter for overexpression in rice. Transgenic *MIR408* overexpression (*MIR408-OE*) lines were generated as before [6]. A ~500-bp genomic fragment, containing the miR408 precursor sequence along with 266 bp of upstream and 253 bp of downstream flanking sequences, was PCR-amplified from Nip genomic DNA and cloned into the pCAMBIA1390 vector, downstream of the *UBI* promoter (S14 Fig). *STTM* structures targeting miR408-3p and miR408-5p were constructed via overlapping PCR, and separately cloned into *pCAMBIA1390* downstream of the *UBI* promoter. The *MIR408-OE* lines (for pre-miR408 overexpression) and *STTM* lines (*STTM-5p* and *STTM-3p*, which specifically impair miR408-5p and miR408-3p activity, respectively) had been characterized previously [6]. To generate the rice mutants *hipp19*, *ucl7*, and *mir408*, we employed the CRISPR-Cas9 system and designed the sgRNA and constructs in line with previously described methodologies [70]. To determine HIPP19 and UCL7 subcellular localization within rice, *GFP-HIPP19* and *GFP-UCL7* fragments were inserted into the pCAMBIA2300 vector to yield the *35S::GFP-HIPP19*/pCAMBIA2300 and *35S::GFP-UCL7*/pCAMBIA2300 constructs. In addition, the *HIPP19* fragment was inserted into the pAN580 vector, resulting in the *35S::HIPP19-GFP/*pAN580 construct. Further refinements included replacing the 35S promoter with the native *HIPP19* and *UCL7* promoters, leading to the creation of the *pro-HIPP19::HIPP19-GFP*/pCAMBIA1305 and *pro-UCL7::UCL7-GFP*/pCAMBIA1305 constructs. For transient transfection assays, we isolated the 500-bp fragment harboring the miR408 or miR530 precursor sequence from rice genomic DNA and inserted it into the pCAMBIA2300 vector under a *CaMV35S* promoter. Additionally, we engineered the *35S::HIPP19-LUC*, *35S::mHIPP19-LUC*, *35S::*UCL7*-LUC*, and *35S::mUCL7-LUC* constructs into the modified pGreen0800 II-LUC vector, incorporating the Renilla (REN) gene to quantify LUC activity relative to internal controls. We transferred all binary expression vectors into *A. tumefaciens* strain AGL1 for rice transformation and strain EHA105 for tobacco leaf infiltration assays. Detailed primer sequences for plasmid construction are listed in S1 Table.

### Quantitative real-time PCR

Nip and the corresponding transgenic lines were exposed to both 0 and 2 μM CdCl_2_ for 12 h, 24 h, and 3 days. Total root RNA was extracted using TRIzol reagent (Invitrogen), followed by DNase I treatment to remove any contaminating DNA. Subsequently, approximately 2 μg of RNA was reverse transcribed into cDNA, which served as the template for Quantitative PCR analysis. qPCR was conducted using SYBR Premix (Invitrogen) on a Real-Time PCR System (Thermo Fisher Scientific), with the *Actin* gene employed as an internal control for normalization. To assess the levels of mature miRNA, stem-loop qRT-PCR was used, as previously described [6], with U6 snRNA serving as the endogenous control. Relative expression levels were calculated using the ΔΔCt (cycle threshold) method. For each qRT-PCR analysis, three biological replicates were conducted. In each biological experiment, samples from three plants (both untreated and Cd-treated groups) were pooled, homogenized, and used for RNA isolation. qRT-PCR was subsequently performed with three technical repeats per sample. The entire procedure was independently replicated three times, and the results are presented as the mean ± standard error. Details of all primer sequences used in this study can be found in S1 Table.

### GUS staining

Transgenic rice seedlings expressing *pro-MIR408-GUS* were subjected to GUS staining to visualize gene expression [6]. The GUS staining solution was composed of 2 mM 5-bromo-4-chloro-3-indolyl-β-D-glucuronic acid, buffered with 50 mM of NaH_2_PO_4_/Na_2_HPO_4_, 2 mM of potassium ferricyanide (K_3_Fe (CN)_6_) and potassium ferrocyanide (K_4_Fe (CN)_6_), 10 mM Na_2_EDTA, and 0.1% Triton X-100. This mixture facilitated the staining process, which took place at an optimal temperature of 37 °C for 8–12 h to achieve clear results. After the GUS staining procedure, we employed 70% ethanol as a medium to efficiently remove chlorophyll, thereby enhancing the visibility of the staining outcomes.

### Luciferase imaging assays

To investigate the influence of miR408-5p on *HIPP19*, we prepared uniform concentrations and volumes of *A.tumefaciens* strain EHA105 containing either wild-type (WT) or mutated target site versions of *HIPP19* in conjunction with *MIR408* or equivalent control vectors. These mixtures were then simultaneously infiltrated into the leaves of *N. benthamiana*. At least five leaves, each from a different *N. benthamiana* specimens underwent infiltration. Following this, the intensity of bioluminescence was quantified using the GloMax Luminometer System (Promega, USA). The results were analyzed based on the LUC/REN ratio, allowing for a quantitative assessment of miR408-5 p’s effects on *HIPP19* expression.

### Degradome dataset analysis

The degradome dataset generated in our study was analyzed as previously described [6]. Briefly, the degradome libraries were constructed from *MIR408-OE* transgenic plants. Degradome library construction, sequencing, and data analysis were essentially described as before [6]. Reads mapping to the predicted *UCL7* target sites were used to determine the positions of the 5′transcript ends using a custom Perl script.

### Subcellular localization and protoplast isolation

To elucidate the subcellular localization of HIPP19 and UCL7, *35S::GFP-HIPP19*, *35S::HIPP19-GFP*, and *35S::UCL7-GFP* constructs were introduced into rice protoplasts using a polyethylene glycol-mediated transformation technique or transiently introduced in the *N. benthamiana* leaf cells, as described before [71,72]. The following transformation, the cells were maintained under dark conditions at room temperature for 15–18 h to facilitate expression. The expression and subcellular localization of the constructs were visualized and captured using a confocal laser scanning microscope (FV1000 MPE; Olympus), providing valuable insights into the cellular distribution and functionality of HIPP19 and UCL7.

### Cell and tissue specificity expression

To investigate the cell and tissue-specific expression patterns of *HIPP19* and *UCL7*, constructs containing the fusion genes *pro-HIPP19::HIPP19-GFP* and *pro-UCL7::UCL7-GFP* were assembled and introduced into *A. tumefaciens* strain AGL1, facilitating the *A. tumefaciens*-mediated transformation of Nip rice. Roots from 10-day-old seedlings of transgenic lines containing the *pro-HIPP19::HIPP19-GFP* construct were selected for detailed immunostaining analysis. The immunostaining procedure, utilizing an anti-GFP antibody according to the methodology described before [73], facilitated the observation of GFP fluorescence under a confocal laser scanning microscope (FV1000 MPE; Olympus). Similarly, transgenic lines harboring the *pro-UCL7::UCL7-GFP* construct were examined using the same confocal microscopy.

### Yeast experiments

The entire open reading frame (ORF) of *HIPP19* and *UCL7* was successfully amplified from cDNA by RT-PCR, using specific primers for each gene. The resultant cDNA fragments were then cloned into the yeast expression vector pYES2, yielding the recombinant constructs *pYES2-HIPP19* and *pYES2-UCL7*. To assess Cd tolerance, the constructs along with the empty vector pYES2 were introduced into the yeast strain SEY6210. Transformants harboring each plasmid were cultured on an SD-uracil solid medium containing 2% galactose and varying concentrations of CdCl_2_ (0, 30, or 40 μM). Growth was documented after incubation at 30 °C for 3 d, and the resulting cultures were photographed. In a parallel set of experiments examining Cd uptake in the liquid culture of yeast (strain SEY6210), each transformant underwent pre-culture overnight in a liquid media formulation complemented with 2% glucose (Glc), 0.67% yeast nitrogen base minus amino acids, and 0.2% drop-out amino acid mix. Following overnight growth, yeast cells were collected by centrifugation and washed thoroughly with Milli-Q water (Millipore). The washed cell pellets were then resuspended in a galactose-containing liquid medium (2% Gal) supplemented with yeast nitrogen base, dropout amino acid mix, and 30 μM CdCl_2_, to conduct the Cd uptake study. The growth of yeast cultures was monitored at distinct time intervals by measuring the optical density at 600 nm (OD600) using a NanoDrop One spectrophotometer (Thermo Scientific, USA). The uptake assay was conducted at 30 °C with horizontal shaking at 200 rpm for 4 h. At the end of the uptake period, the cells were washed three times with Milli-Q water. The dry weight of the yeast pellets was determined after a drying period of 48 h at 65 °C. Subsequently, dried yeast biomass was subjected to 65% HNO_3_ digestion for the subsequent quantification of acquired Cd. Each experimental condition was independently replicated at least three times to ensure reproducibility of the results.

### Protein purification and metal binding assay

To directly assess potential metal-binding properties, the full-length coding sequences of *HIPP19* and *UCL7* were amplified from cDNA using RT-PCR, employing specific primers, as detailed in S1 Table. These amplified fragments were then cloned into the vector pMAL-C2X. Subsequently, the constructs were introduced into the *E. coli* strain Rosette (DE3) for expression. After confirming the presence of the inserted sequences, a positive strain was cultured in Luria-Bertani (LB) liquid medium (5 mL) supplemented with 50 mg/L ampicillin and incubated overnight at 37 °C with agitation at 180 rpm. The overnight cultures (2.5 mL each) were then transferred into 250 mL of LB medium to facilitate further growth. Upon reaching an OD600 of 0.6, expression of the recombinant proteins was induced with 0.5 mM isopropyl β-D-1-thiogalactopyranoside (IPTG) and maintained at 25 °C for 12 h before harvesting the cells. Protein purification was performed in accordance with the MBP protein purification protocol provided by Smart-lifesciences, with slight modifications. The cell cultures were lysed by sonication, and the resulting MBP-HIPP19 and MBP-UCL7 fusion proteins were then incubated with 10 μM Cd and Cu in 25 mL PBS buffer (comprising 0.02% KCl, 0.8% NaCl, 0.144% Na_2_HPO_4_, and 0.024% KH_2_PO_4_, at pH 7.0) for 4 h at 4 °C. Proteins were eluted with a solution containing 10 mM maltose, 20 mM Tris-HCl, and 1 mM EDTA. Before ICP-MS analysis, the purified proteins were digested with 65% HNO3 at 120 °C for 2 h. The concentrations of the purified proteins were quantified using a BCA protein assay kit supplied by Beyotime. To guarantee the reliability and repeatability of our results, each experiment was independently conducted at least three times, following the methodologies outlined [74].

### H_2_O_2_ and SOD measurements

Hydrogen peroxide (H_2_O_2_) levels in the roots of 2-week-old rice seedlings were determined using a precise methodology. Initially, fresh rice roots were harvested and pulverized into a fine powder under liquid nitrogen. To this powder, lysate, and sodium phosphate buffer were added, followed by a 30-min incubation on ice. The resultant mixture was then centrifuged at 12,000*g* and 4 °C for 20 min. The concentration of H_2_O_2_ in the supernatant was accurately measured using a hydrogen peroxide assay kit (Beyotime) according to the instructions provided by the manufacturer. The Optical Density (O.D.) at 450 nm was read using a microtiter plate reader (BioTek Microplate Reader). This standard curve was employed to quantify the H_2_O_2_ concentration in an unknown sample. The activity of SOD was determined using the Plant Super Oxidase Dimutase ELISA Kit (MEIMIAN), ensuring a comprehensive analysis of oxidative stress responses in rice seedling roots. The specific ROS fluorescence H_2_O_2_ Probe (MKBio, China) were used to visualize H_2_O_2_ in protoplasts and root cells, respectively. This approach follows the methodologies described before [75,76]. The relative fluorescence intensity was quantified using ImageJ software.

### Transfection of rice protoplasts and western blots

Generally, the plasmids *35S-Flag-HIPP19-CDS* or *35S-Flag-HIPP19-mCDS* were transformed into rice protoplasts according to previously described protocols [72]. Briefly, protoplasts were isolated from 10-day-old WT and the indicated transgenic rice seedlings, which were either untreated or treated with 2 μM CdCl_2_ for 24 hours. Around 100 μL protoplast suspension (containing ~2 × 10^5^ protoplasts) was transfected with 2 μg of plasmid in a 120 μL PEG solution. The transformation mixture was incubated in the dark for 15 min at 28 °C, then diluted with 1 mL W5 solution (NaCl, 154 mM; CaCl_2_, 125 mM; KCl, 5 mM; MES, 2 mM, pH 5.7), and centrifuged at 120g for 3 min. Protoplasts were suspended in W1 solution (Mannitol, 0.5 M; KCl, 20 mM; MES, 4 mM, pH 5.7), transferred into 2 mL centrifuge tubes, and incubated at 28 °C for 16 h. Total Proteins were extracted from half of the transfected protoplasts, and the remaining protoplasts were used for RNA extraction and RT-PCR.

For immunoblot analyses, total proteins were isolated from the corresponding protoplasts of WT and transgenic seedlings. Proteins were separated by sodium dodecyl sulfate-polyacrylamide gel electrophoresis, transferred to polyvinylidene difluoride membranes, immunoblotted with corresponding commercial antibodies, and detected using High-sig ECL Western Blotting Substrate (Tanon). Quantification of immunoblots was performed according to the band intensities of Flag-HIPP19 and GFP as a control, which were measured using ImageJ software. Relative band intensities were then calculated using the ratio of HIPP19/GFP for each immunoblot. All immunoblot experiments were performed at least three times, with the same conclusions.

### Determination of metal concentration and translocation

Rice samples were washed six times with EDTA solution to remove extracellularly bound Cd, dried at 65 °C, and then digested in 70% nitric acid at 120 °C for 4 hours, as previously described [77–79]. Samples were diluted with Millipore-filtered deionized water. The metal contents were quantified using inductively coupled plasma mass spectrometry (ICP-MS). The measurement of metal concentration was performed at least five biological replicates, with each replicate comprising a mixture of 3 plants. The rate of Cd translocation from the root to the shoot was estimated by calculating the percentage of Cd in the shoot relative to the entire plant [56].

## Supporting information

S1 Fig The involvement of *MIR408* in Cd uptake in rice. **(A)** Time course analysis of pre-miR408 expressions in 14 d-old Nipponbare (Nip) plants with 10 μM CdCl_2_ treatment. *Actin* was used as an internal control for the normalization of the qRT-PCR results. Values are means ± SD (*n* = 3 biological replicates). **(B)** and **(C)** The contents of Mn, Fe, Cu, and Zn in roots (B) and shoots (C) of 14 d-old WT and *MIR408-OE* seedlings grown in 2 μM CdCl_2_ conditions. Values are means ± SD (n = 5 biological replicates). **(D)** and **(E)** The contents of Mn, Fe, Cu, and Zn in roots (D) and shoots (E) of 14 d-old WT and *mir408* mutants grown in 2 μM CdCl_2_ conditions. Error bars indicate SD (Tukey’s honestly signiﬁcant difference, *P < 0.05). Values are means ± SD (*n* = 5 biological replicates). The data underlying this Figure can be found in S1 Data.
(TIFF)

S2 Fig miR408-5p regulates *HIPP19* in a translation repression manner. **(A)** Time course analysis of mature miR408-5p accumulation in 14 d-old Nip plants with 10 μM CdCl_2_ treatment (Student’s *t* test, ***P < 0.001). Values are means ± SD (*n* = 3 biological replicates). **(B)** Gene structure of *HIPP19* and alignments of miR408-5p with target sites in *HIPP19* CDS and the indicated mutant CDS (m2CDS). **(C)** Relative expressions of *HIPP19* in 14 d-old Nip plants with different time of 10 μM CdCl_2_ treatment. Values are means ± SD (n = 3 biological replicates). **(D)** Validation of *HIPP19* as miR408-5p target through transient expression analysis in *N. benthamiana* leaves. Left: The constructs in *A. tumefaciens* transiently introduced in *N. benthamiana* leaves. Middle: Representative photograph of ﬁreﬂy luciferase ﬂuorescence signals when the indicated construct combinations were introduced in *N. benthamiana* leaves. Right: Relative reporter activity in *N. benthamiana* leaves expressing the indicated construct combinations. Error bars indicate SD (Tukey’s honestly signiﬁcant difference, *P* < 0.05) (*n* = 3 biological replicates). **(E)** Relative expressions of *UCL8* in WT and *mir408* mutants. Values are means ± SD (*n* = 3 biological replicates). **(F)** Relative expressions of *HIPP19* in WT, *STTM-5p* and *STTM-3p* plants with or without 2 μM Cd treatment for 24 h. ns, not signiﬁcant (Student’s *t* tes*t*). Values are means ± SD (*n* = 3 biological replicates). **(G)** Relative accumulation of miR408-5p in WT, *STTM-5p* and *STTM-3p* plants with or without 2 μM Cd treatment (Student’s *t* tes*t*, ***P < 0.001). Values are means ± SD (*n* = 3 biological replicates). **(H)** The protein and mRNA levels of HIPP19 in independent *HIPP19* and *HIPP19m* transgenic plants, with or without 2 μM Cd treatment. HIPP19 protein was detected using an anti-GFP antibody, and its mRNA was assessed by RT-PCR analysis of the P2 band corresponding to the fragment shown in Fig 2J. Actin protein and mRNA levels served as loading controls. (I) The relative protein level of HIPP19 when the cassettes of constructs shown in Fig 2F were introduced into rice protoplasts isolated from WT, *STTM-5p,* and *STTM-3p* plants grown with or without 2 μM CdCl_2_ treatment. The experiments were performed three times and one of the representative results was shown below the columns. The data underlying this Figure can be found in S1 Data and S1 Raw Images.
(TIFF)

S3 Fig Generation of *hipp19* mutants. Sequences of CRISPR-cas9 alleles of *hipp19* mutants. sgRNA and PAM sequences were marked by black lines and red colors, respectively.
(TIFF)

S4 Fig HIPP19 is involved in short-term Cd uptake in rice. **(A)** The contents of Cd in roots and shoots of 14 d-old WT and *hipp19* mutants grown in 2 μM CdCl_2_ conditions for 30 min (Student’s *t* test, *P < 0.01;**P < 0.01; ***P < 0.001). Values are means SD (*n* = 8 biological replicates). **(B)** Translocation of Cd from roots to shoots of WT and *hipp19* mutants exposed in 2 μM CdCl_2_ for 30 min. ns, not signiﬁcant (Student’s *t* test). **(C)** The contents of Cd in roots and shoots of 14 d-old WT and *hipp19* mutants grown in 2 μM CdCl_2_ conditions for 2 hours. (Student’s *t* test, ***P < 0.001). Values are means ± SD (*n* = 8 biological replicates). **(D)** Translocation of Cd from roots to shoots of WT and *hipp19* mutants exposed in 2 μM CdCl_2_ for 2 hours. ns, not signiﬁcant (Student’s *t* test). The data underlying this Figure can be found in S1 Data.
(TIFF)

S5 Fig Analysis of *mir408*/*hipp19* double mutants and HIPP19 sub-localization. **(A)** Sequences of CRISPR-cas9 alleles of *hipp19*/*mir408* mutants. sgRNA and PAM sequences were marked by black lines and red colors, respectively. **(B)** Subcellular location analysis of HIPP19-GFP and GFP-HIPP19 in *N. benthamiana* leaf cells. PIP2a-mCherry and NLS-CFP represents the localization in plasma membrane and nucleus, respectively. Bars = 50 μm.
(TIFF)

S6 Fig UCL7 is involved in Cd uptake regulation in rice. **(A)** Time course analysis of miR408-3p accumulations in Nip plants with 10 μM CdCl_2_ treatment (Student’s *t* test, *P < 0.01,**P < 0.01, ***P < 0.001). Values are means ± SD (n = 3 biological replicates). **(B)** Expression analysis of different *UCL* family member genes in Nip plants with 10 μM CdCl_2_ treatment. Values are means ± SD (*n* = 3 biological replicates). **(C)** Relative expressions of *UCL7* in WT, STTM-5p and STTM-3p plants with or without 24 h 2 μM Cd treatment (Student’s *t* test, ***P < 0.001). Values are means ± SD (*n* = 3 biological replicates). **(D)** Relative accumulation of miR408-3p in WT, *STTM-5p* and *STTM-3p* plants with or without 2 μM Cd treatment. Values are means ± SD (*n* = 3 biological replicates). **(E)** The contents of Fe, Mn, Cu, and Zn in roots and shoots of 14 d-old WT and STTM-3p plants grown in 2 μM CdCl_2_ conditions (Tukey’s honestly signiﬁcant difference, *P* < 0.05). Values are means ± SD (*n* = 5 biological replicates). **(F)** Sequences of CRISPR-cas9 alleles of *ucl7* mutants. **(G)** The contents of Fe, Mn, Cu, and Zn in roots and shoots of 14 d-old WT and *ucl7* mutants grown in 2 μM CdCl_2_ conditions (Tukey’s honestly signiﬁcant difference, *P* < 0.05). Values are means ± SD (*n* = 5 biological replicates). The data underlying this Figure can be found in S1 Data.
(TIFF)

S7 Fig UCL7 plays a role in repressing Cd absorption in rice. **(A)** Dilution-series spot assays of yeast strain SEY6210 growth expressing *UCL7*, *Nramp5,* or empty vector YES2 in a medium containing the indicated concentrations of Cd. **(B)** Growth of yeast strains shown in (A) with or without different times of 30 μM CdCl_2_ treatment. **(C)** Cd concentrations in yeast cells expressing *UCL7*, *Nramp5,* or empty vector YES2 after incubation in a liquid medium containing 30 μM Cd for 12 h (Student’s *t* test, ***P < 0.001). Values are means ± SD (*n* = 3 biological replicates). **(D)** In vitro metal ion binding assay of UCL7 to Cu and Cd. Full-length UCL7 recombinant proteins were extracted from BL21 and then incubated with 10 µM Cu and 10 µM Cd for 1 h, pH = 7.4. MBP represents the *E. coli* trigger factor protein that fused to the N-terminus of the indicated proteins. GFP protein was used as a control (Student’s *t* test, ***P < 0.001). Values are means ± SD (*n* = 3 biological replicates). The data underlying this Figure can be found in S1 Data.
(TIFF)

S8 Fig *hipp19* and *ucl7* mutants have no negative effects on rice major agronomic traits. **(A)** The plant architecture of *hipp19* and *ucl7* mutants grown in soil under open-field natural conditions for 4 months. Bars = 10 cm. **(B)** The plant height, number of effective panicles per plant, number of grains per panicle, and 1,000-grain weight of *hipp19* mutants. ns, not signiﬁcant (Student’s *t* test). Values are means ± SD (*n* = 10 independent plants). **(C)** The plant height, number of effective panicles per plant, number of grains per panicle, and 1,000-grain weight of *ucl7* mutants. ns, not signiﬁcant (Student’s *t* test). Values are means ± SD (*n* = 10 independent plants). The data underlying this Figure can be found in S1 Data.
(TIFF)

S9 Fig Rice HIPP19 and Nramp5 cannot physically interact. Left: The constructs in *A. tumefaciens* transiently introduced in *N. benthamiana* leaves in Firefly luciferase complementation imaging (FLCI) assays. Middle: Representative photograph of ﬁreﬂy luciferase ﬂuorescence signals when the indicated construct combinations were introduced in *N. benthamiana* leaves. Right: Relative LUC activity in *N. benthamiana* leaves expressing the indicated construct combinations. The known interaction between FT1 and FD1 was used as a positive control. Error bars indicate SD (*n* = 8 biological replicates) (Tukey’s honestly signiﬁcant difference, *P* < 0.05). The data underlying this Figure can be found in S1 Data.
(TIFF)

S10 Fig Conjectural working models for the regulation of Cd uptake and accumulation in rice involving HIPP19. In WT plants, plasma membrane-localized HIPP19 likely facilitates Cd uptake into cells, possibly with or without the assistance of an unknown Cd transporter. Additionally, cytoplasmic HIPP19 may bind Cd²⁺, buffering free Cd²⁺ levels in the cytosol and potentially triggering feedback regulation of heavy metal-sensing pathways. In *MIR408-OE* plants, elevated miR408-5p represses HIPP19 protein accumulation, thereby disrupting Cd uptake. Purple clumps represent HIPP19 protein and blue barrel-shaped structures represent transporters.
(TIFF)

S11 Fig A phylogenetic tree of HIPP family proteins in rice. (TIFF)

S12 Fig Conjectural working models for the regulation of Cd uptake and accumulation in rice involving UCL7. In WT plants, UCL7 may suppress SOD activity, resulting in low H_2_O_2_ production and consequently high Cd uptake. In *MIR408-OE* plants, elevated miR408-3p enhances cleavage of UCL7 transcripts, reducing the pool of UCL7 mRNA available for translation. As cytoplasmic UCL7 protein binds Cu²⁺, its depletion may increase intracellular copper availability, thereby boosting Cu/Zn SOD activity. These sequential events likely promote localized H_2_O_2_ production and the enhanced H_2_O_2_ accumulation in roots and shoots may modulate cation channel activity or alter heavy metal-related protein modifications, ultimately inhibiting Cd uptake. Green structures indicate UCL7 protein, gray structures denote SOD enzymes, black dots represent Cd²⁺ ions, and yellow dots represent Cu²⁺ ions.
(TIFF)

S13 Fig miR398 is induced by 2 μM Cd treatment in rice. Student’s *t* test. ***P < 0.001. Values are means ± SD (*n* = 3 biological replicates). The data underlying this Figure can be found in S1 Data.
(TIFF)

S14 Fig Clarification of the fragments that are overexpressed in *MIR408OE* and detected by qRT-PCR in this study. The upper sequences and lower lines highlighted in green represent the miR408 precursor sequence and its corresponding location, respectively. The upper sequences and lower lines marked in blue denote the primers used for detecting *MIR408* expression and their respective positions, respectively. The upper sequences and lower lines marked in red represent the primers used for *MIR408* overexpression and their respective positions, respectively.
(TIFF)

S1 Table The Oligos used in this study. (XLSX)

S1 Data The data underlying Figures. (XLSX)

S1 Raw Images The uncropped images underlying Figures. (PDF)

## Abbreviations

DW: dry weight
HIPP: heavy metal-associated isoprenylated plant protein
HMA: heavy metal-associated domain
IPTG: isopropyl β-D-1-thiogalactopyranoside
LB: Luria-Bertani
LUC: luciferase
miRNA: microRNA
Nip: Nipponbare
NRAMP: natural resistance-associated macrophage protein
ORF: open reading frame
PCs: phytochelates
PCR: polymerase chain reaction
REN: Renilla
ROS: reactive oxygen species
SODs: superoxide dismutases
STTM-5p: Small Tandem Target Mimic-5p
UBI: ubiquitin
UCL7: Uclacyanin 7
WT: wild-type
