# Supplementary figures and images for: miR408-5p and miR408-3p cooperatively reduce cadmium uptake and accumulation in rice
Source: PLoS Biol. 2026 Jun 2;24(6):e3003811. doi: 10.1371/journal.pbio.3003811 (PMC13229367; doi:10.1371/journal.pbio.3003811)

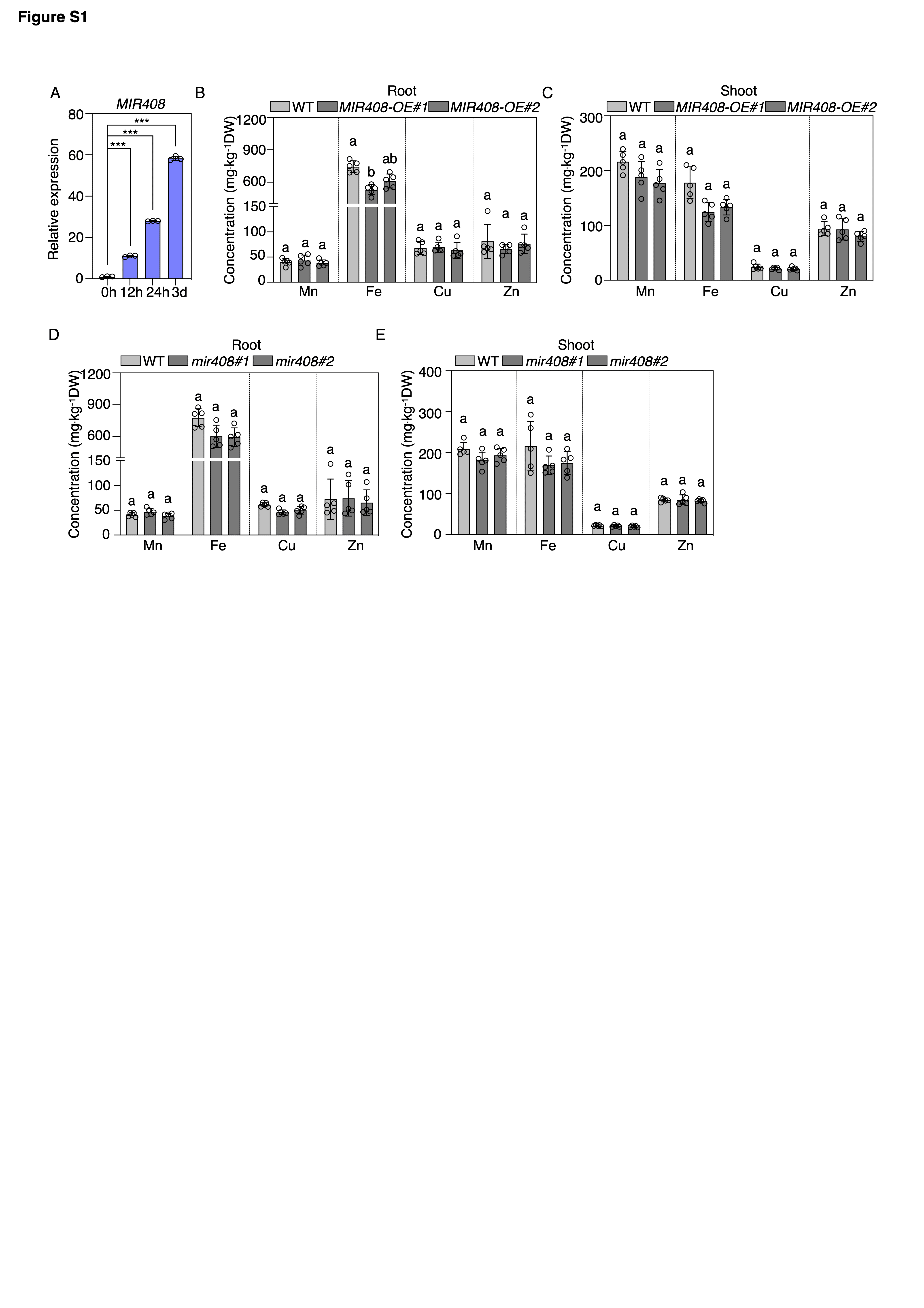

Supplement: S1 Fig — (A) Time course analysis of pre-miR408 expressions in 14 d-old Nipponbare (Nip) plants with 10 μM CdCl2 treatment. Actin was used as an internal control for the normalization of the qRT-PCR results. Values are means ± SD (n = 3 biological replicates). (B) and (C) The contents of Mn, Fe, Cu, and Zn in roots (B) and shoots (C) of 14 d-old WT and MIR408-OE seedlings grown in 2 μM CdCl2 conditions. Values are means ± SD (n = 5 biological replicates). (D) and (E) The contents of Mn, Fe, Cu, and Zn in roots (D) and shoots (E) of 14 d-old WT and mir408 mutants grown in 2 μM CdCl2 conditions. Error bars indicate SD (Tukey’s honestly signiﬁcant difference, *P < 0.05). Values are means ± SD (n = 5 biological replicates). The data underlying this Figure can be found in S1 Data. (TIFF) [file pbio.3003811.s001.tiff]

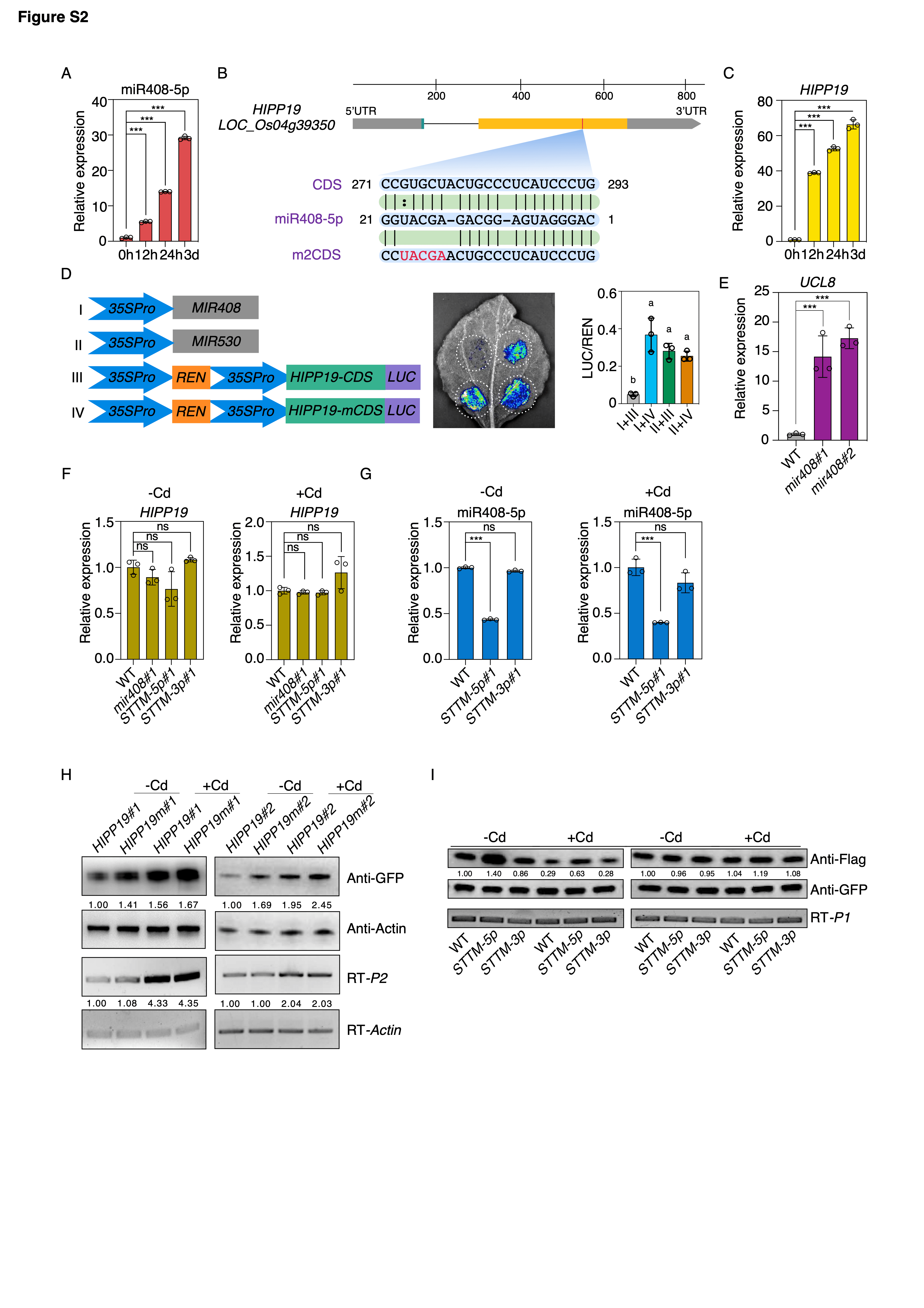

Supplement: S2 Fig — (A) Time course analysis of mature miR408-5p accumulation in 14 d-old Nip plants with 10 μM CdCl2 treatment (Student’s t test, ***P < 0.001). Values are means ± SD (n = 3 biological replicates). (B) Gene structure of HIPP19 and alignments of miR408-5p with target sites in HIPP19 CDS and the indicated mutant CDS (m2CDS). (C) Relative expressions of HIPP19 in 14 d-old Nip plants with different time of 10 μM CdCl2 treatment. Values are means ± SD (n = 3 biological replicates). (D) Validation of HIPP19 as miR408-5p target through transient expression analysis in N. benthamiana leaves. Left: The constructs in A. tumefaciens transiently introduced in N. benthamiana leaves. Middle: Representative photograph of ﬁreﬂy luciferase ﬂuorescence signals when the indicated construct combinations were introduced in N. benthamiana leaves. Right: Relative reporter activity in N. benthamiana leaves expressing the indicated construct combinations. Error bars indicate SD (Tukey’s honestly signiﬁcant difference, P < 0.05) (n = 3 biological replicates). (E) Relative expressions of UCL8 in WT and mir408 mutants. Values are means ± SD (n = 3 biological replicates). (F) Relative expressions of HIPP19 in WT, STTM-5p and STTM-3p plants with or without 2 μM Cd treatment for 24 h. ns, not signiﬁcant (Student’s t test). Values are means ± SD (n = 3 biological replicates). (G) Relative accumulation of miR408-5p in WT, STTM-5p and STTM-3p plants with or without 2 μM Cd treatment (Student’s t test, ***P < 0.001). Values are means ± SD (n = 3 biological replicates). (H) The protein and mRNA levels of HIPP19 in independent HIPP19 and HIPP19m transgenic plants, with or without 2 μM Cd treatment. HIPP19 protein was detected using an anti-GFP antibody, and its mRNA was assessed by RT-PCR analysis of the P2 band corresponding to the fragment shown in Fig 2J. Actin protein and mRNA levels served as loading controls. (I) The relative protein level of HIPP19 when the cassettes of constructs shown in Fig 2F w [file pbio.3003811.s002.tiff]

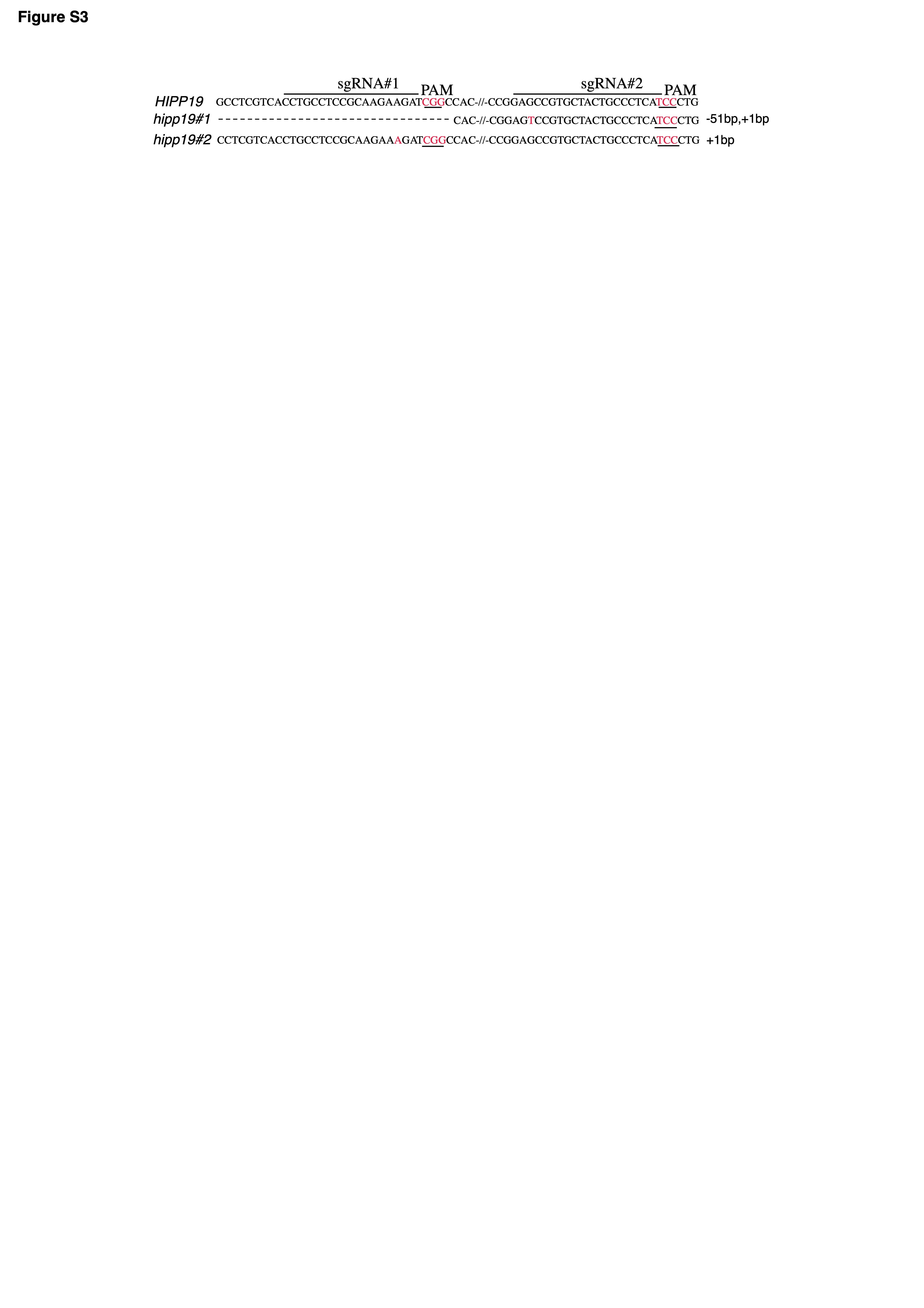

Supplement: S3 Fig — Sequences of CRISPR-cas9 alleles of hipp19 mutants. sgRNA and PAM sequences were marked by black lines and red colors, respectively. (TIFF) [file pbio.3003811.s003.tiff]

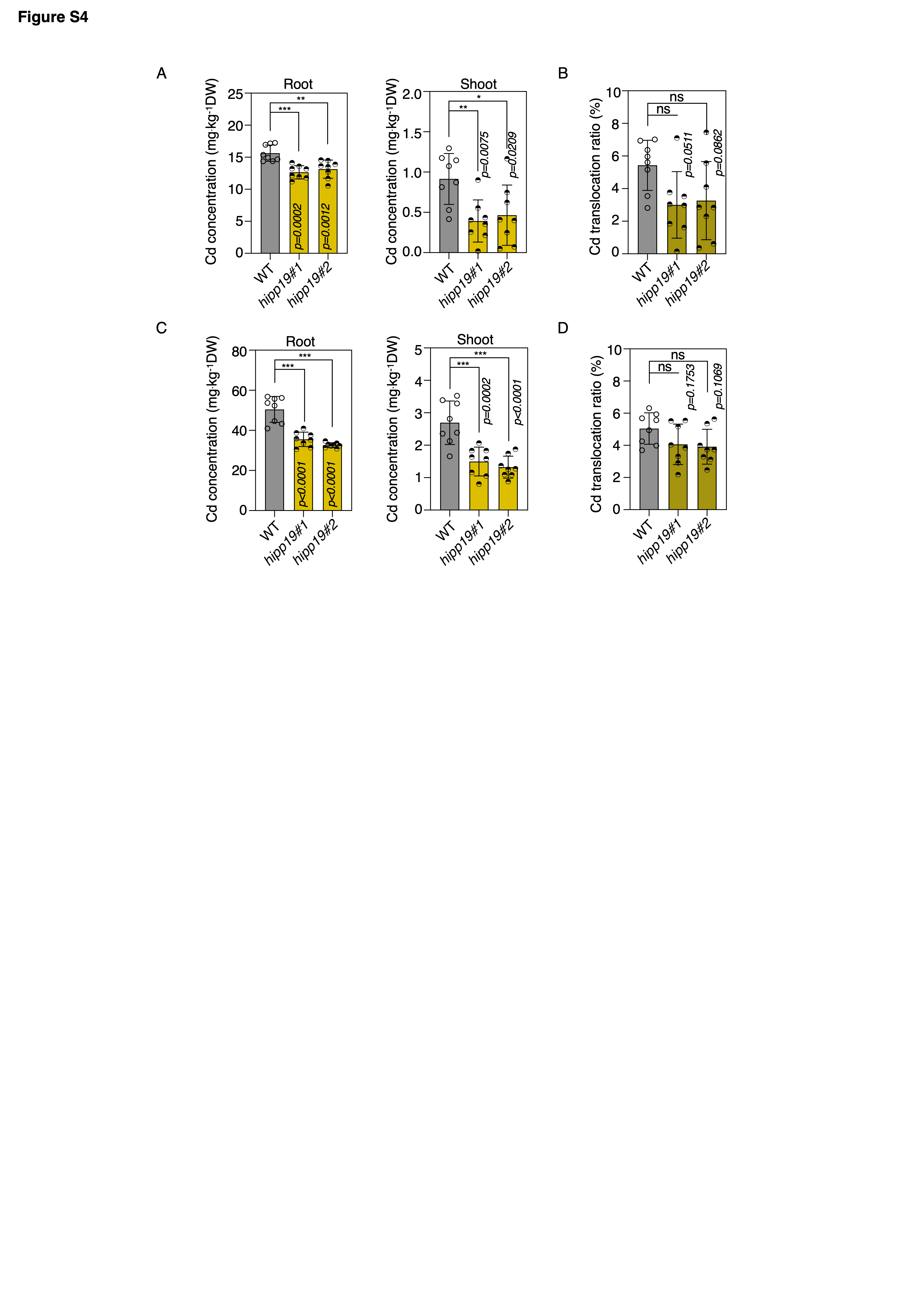

Supplement: S4 Fig — (A) The contents of Cd in roots and shoots of 14 d-old WT and hipp19 mutants grown in 2 μM CdCl2 conditions for 30 min (Student’s t test, *P < 0.01;**P < 0.01; ***P < 0.001). Values are means SD (n = 8 biological replicates). (B) Translocation of Cd from roots to shoots of WT and hipp19 mutants exposed in 2 μM CdCl2 for 30 min. ns, not signiﬁcant (Student’s t test). (C) The contents of Cd in roots and shoots of 14 d-old WT and hipp19 mutants grown in 2 μM CdCl2 conditions for 2 hours. (Student’s t test, ***P < 0.001). Values are means ± SD (n = 8 biological replicates). (D) Translocation of Cd from roots to shoots of WT and hipp19 mutants exposed in 2 μM CdCl2 for 2 hours. ns, not signiﬁcant (Student’s t test). The data underlying this Figure can be found in S1 Data. (TIFF) [file pbio.3003811.s004.tiff]

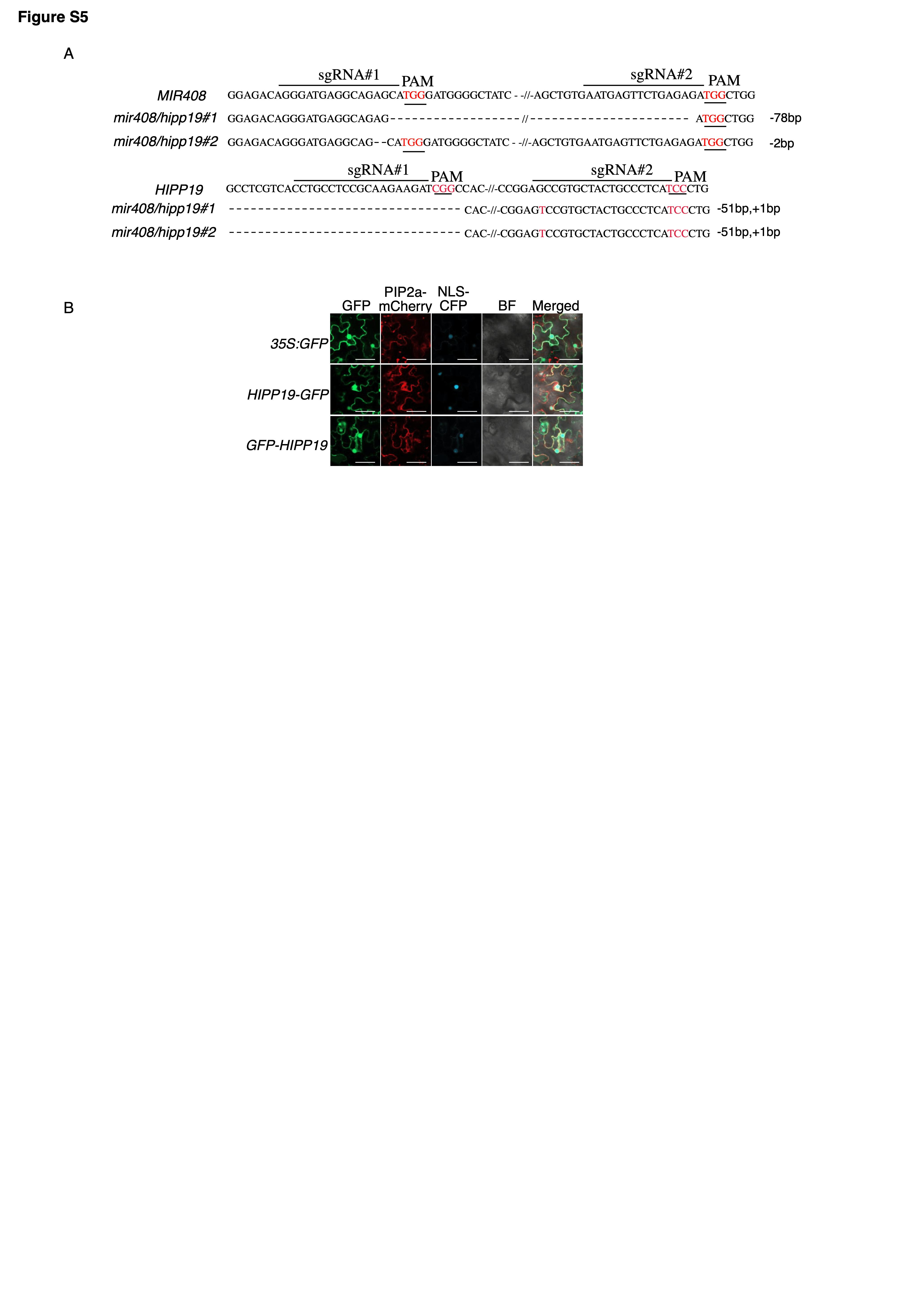

Supplement: S5 Fig — (A) Sequences of CRISPR-cas9 alleles of hipp19/mir408 mutants. sgRNA and PAM sequences were marked by black lines and red colors, respectively. (B) Subcellular location analysis of HIPP19-GFP and GFP-HIPP19 in N. benthamiana leaf cells. PIP2a-mCherry and NLS-CFP represents the localization in plasma membrane and nucleus, respectively. Bars = 50 μm. (TIFF) [file pbio.3003811.s005.tiff]

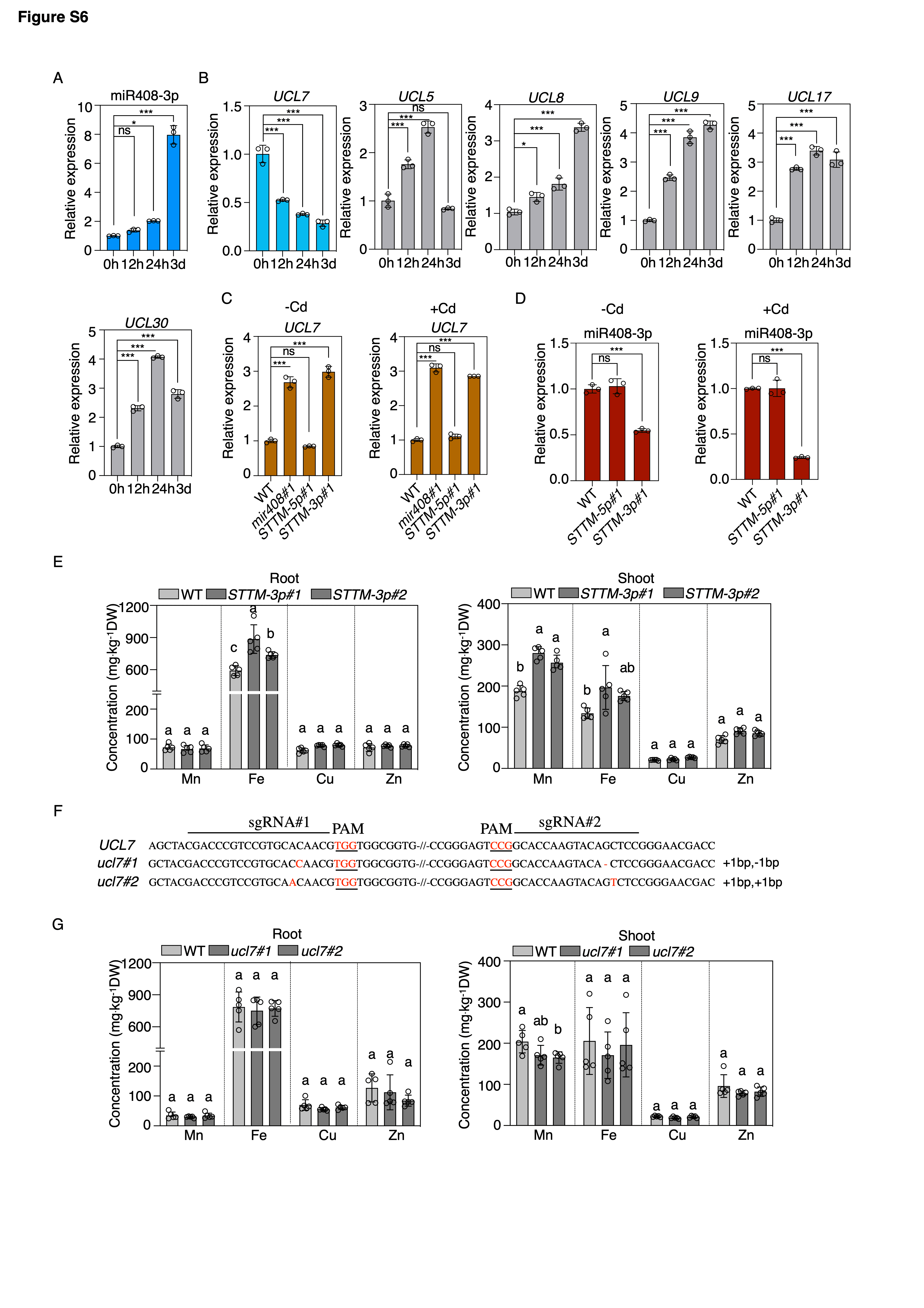

Supplement: S6 Fig — (A) Time course analysis of miR408-3p accumulations in Nip plants with 10 μM CdCl2 treatment (Student’s t test, *P < 0.01,**P < 0.01, ***P < 0.001). Values are means ± SD (n = 3 biological replicates). (B) Expression analysis of different UCL family member genes in Nip plants with 10 μM CdCl2 treatment. Values are means ± SD (n = 3 biological replicates). (C) Relative expressions of UCL7 in WT, STTM-5p and STTM-3p plants with or without 24 h 2 μM Cd treatment (Student’s t test, ***P < 0.001). Values are means ± SD (n = 3 biological replicates). (D) Relative accumulation of miR408-3p in WT, STTM-5p and STTM-3p plants with or without 2 μM Cd treatment. Values are means ± SD (n = 3 biological replicates). (E) The contents of Fe, Mn, Cu, and Zn in roots and shoots of 14 d-old WT and STTM-3p plants grown in 2 μM CdCl2 conditions (Tukey’s honestly signiﬁcant difference, P < 0.05). Values are means ± SD (n = 5 biological replicates). (F) Sequences of CRISPR-cas9 alleles of ucl7 mutants. (G) The contents of Fe, Mn, Cu, and Zn in roots and shoots of 14 d-old WT and ucl7 mutants grown in 2 μM CdCl2 conditions (Tukey’s honestly signiﬁcant difference, P < 0.05). Values are means ± SD (n = 5 biological replicates). The data underlying this Figure can be found in S1 Data. (TIFF) [file pbio.3003811.s006.tiff]

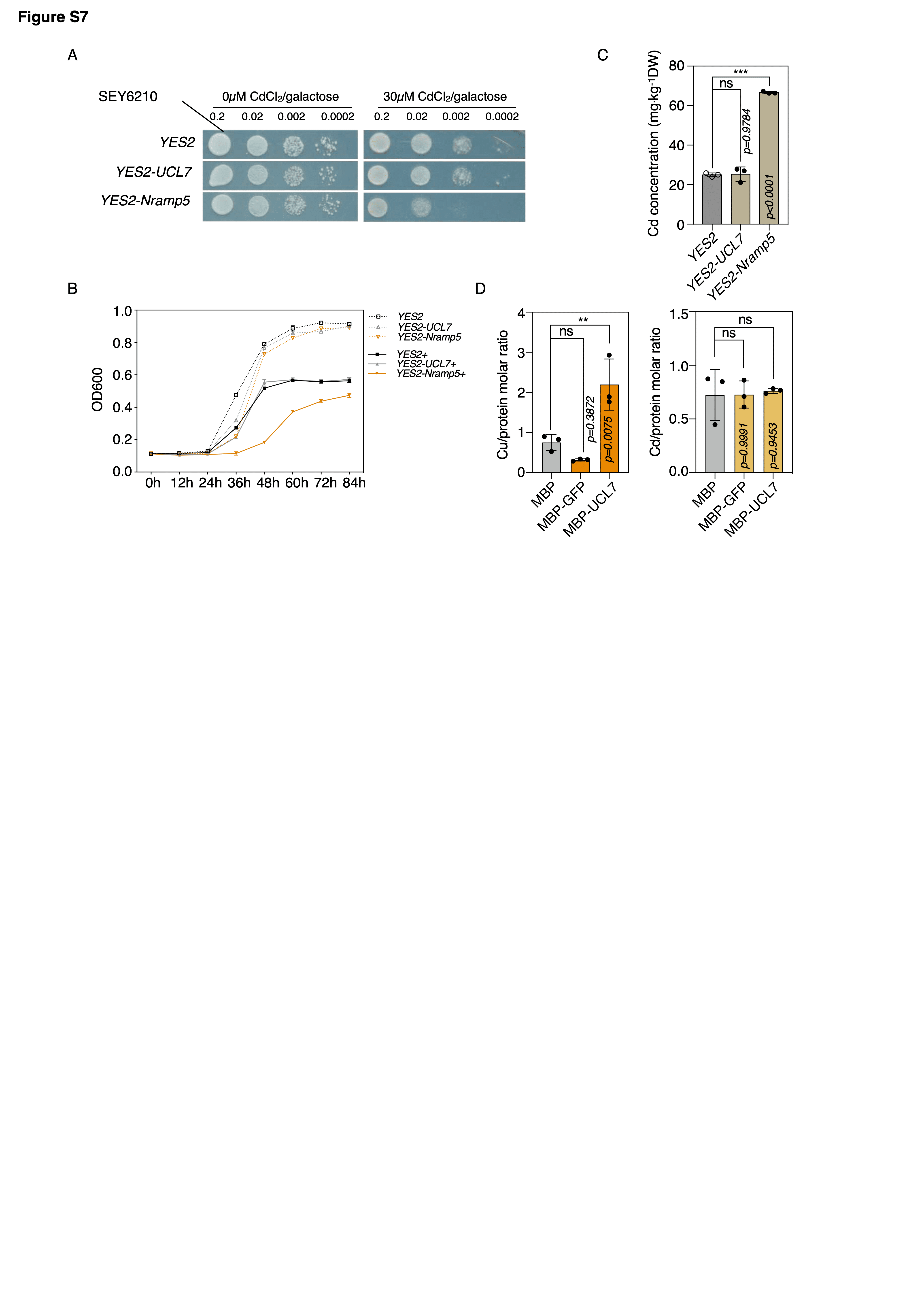

Supplement: S7 Fig — (A) Dilution-series spot assays of yeast strain SEY6210 growth expressing UCL7, Nramp5, or empty vector YES2 in a medium containing the indicated concentrations of Cd. (B) Growth of yeast strains shown in (A) with or without different times of 30 μM CdCl2 treatment. (C) Cd concentrations in yeast cells expressing UCL7, Nramp5, or empty vector YES2 after incubation in a liquid medium containing 30 μM Cd for 12 h (Student’s t test, ***P < 0.001). Values are means ± SD (n = 3 biological replicates). (D) In vitro metal ion binding assay of UCL7 to Cu and Cd. Full-length UCL7 recombinant proteins were extracted from BL21 and then incubated with 10 µM Cu and 10 µM Cd for 1 h, pH = 7.4. MBP represents the E. coli trigger factor protein that fused to the N-terminus of the indicated proteins. GFP protein was used as a control (Student’s t test, ***P < 0.001). Values are means ± SD (n = 3 biological replicates). The data underlying this Figure can be found in S1 Data. (TIFF) [file pbio.3003811.s007.tiff]

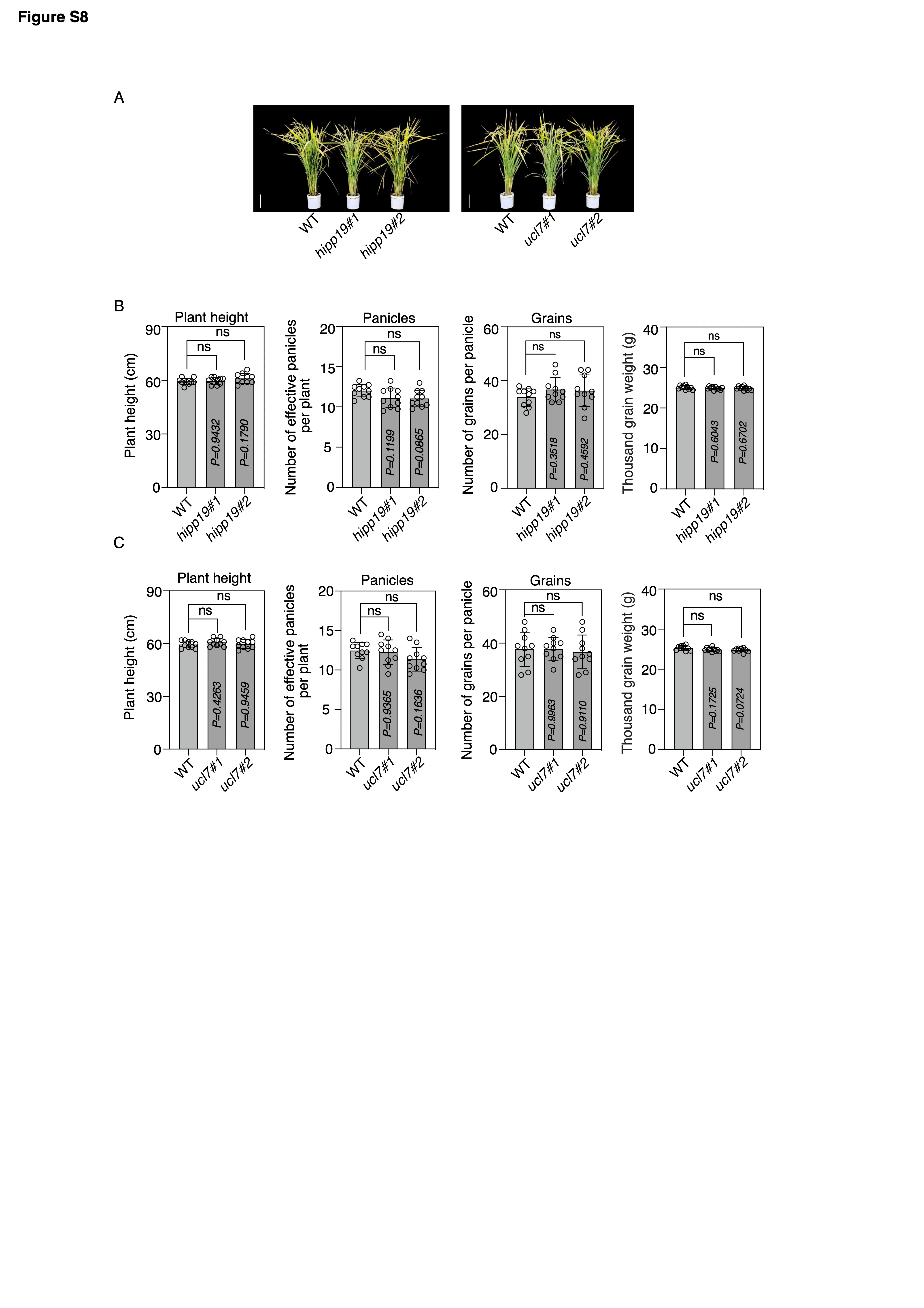

Supplement: S8 Fig — (A) The plant architecture of hipp19 and ucl7 mutants grown in soil under open-field natural conditions for 4 months. Bars = 10 cm. (B) The plant height, number of effective panicles per plant, number of grains per panicle, and 1,000-grain weight of hipp19 mutants. ns, not signiﬁcant (Student’s t test). Values are means ± SD (n = 10 independent plants). (C) The plant height, number of effective panicles per plant, number of grains per panicle, and 1,000-grain weight of ucl7 mutants. ns, not signiﬁcant (Student’s t test). Values are means ± SD (n = 10 independent plants). The data underlying this Figure can be found in S1 Data. (TIFF) [file pbio.3003811.s008.tiff]

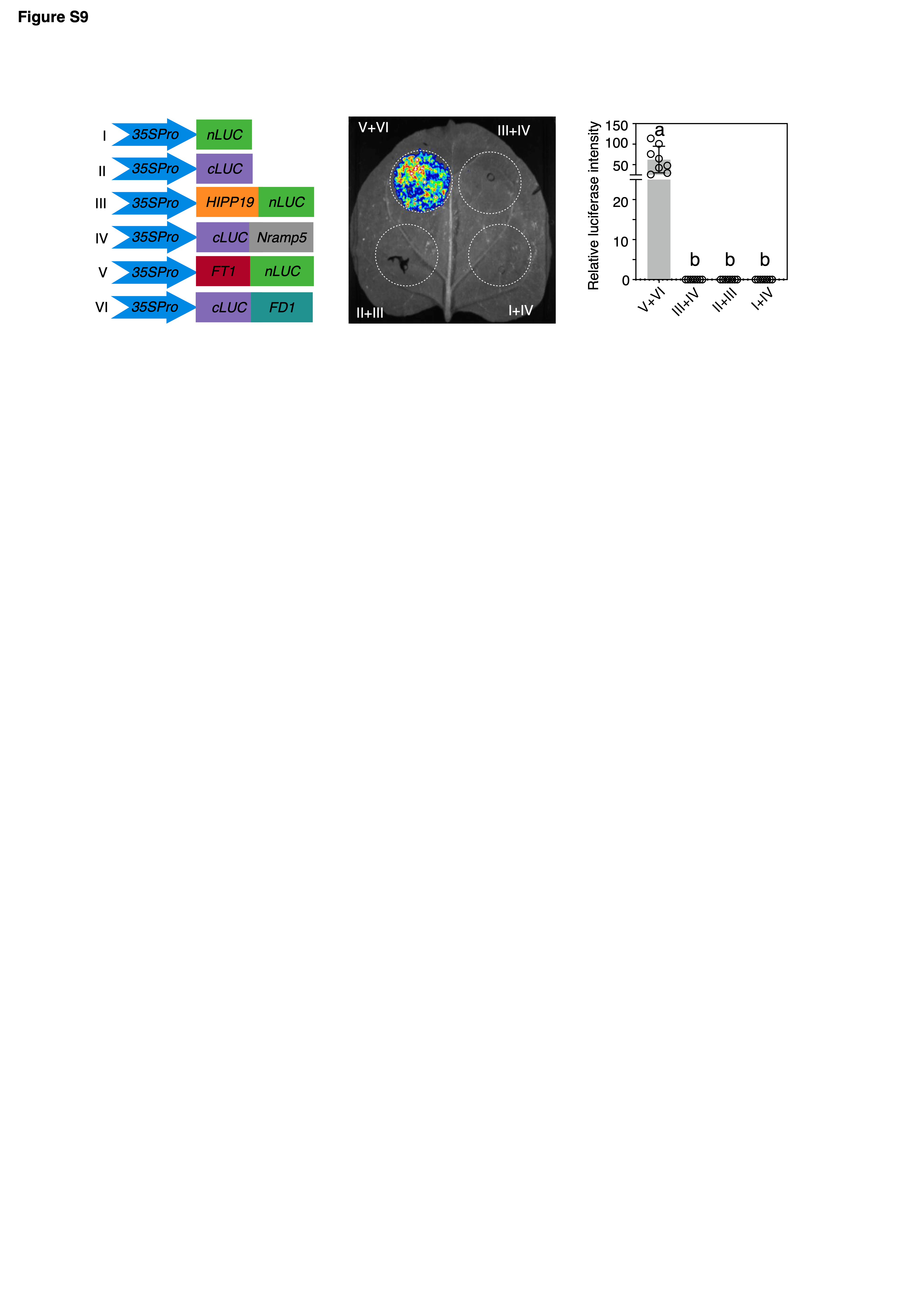

Supplement: S9 Fig — Left: The constructs in A. tumefaciens transiently introduced in N. benthamiana leaves in Firefly luciferase complementation imaging (FLCI) assays. Middle: Representative photograph of ﬁreﬂy luciferase ﬂuorescence signals when the indicated construct combinations were introduced in N. benthamiana leaves. Right: Relative LUC activity in N. benthamiana leaves expressing the indicated construct combinations. The known interaction between FT1 and FD1 was used as a positive control. Error bars indicate SD (n = 8 biological replicates) (Tukey’s honestly signiﬁcant difference, P < 0.05). The data underlying this Figure can be found in S1 Data. (TIFF) [file pbio.3003811.s009.tiff]

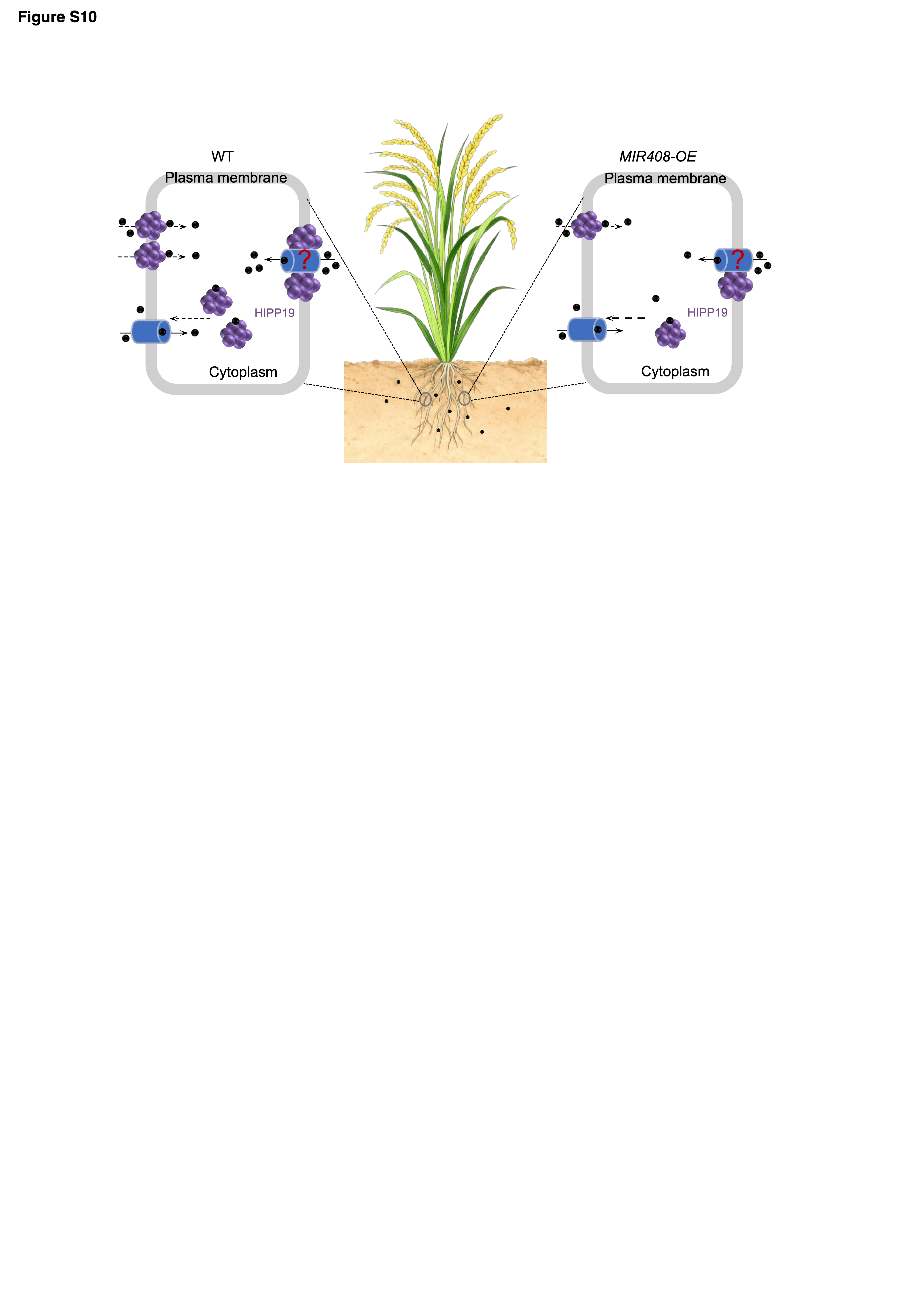

Supplement: S10 Fig — In WT plants, plasma membrane-localized HIPP19 likely facilitates Cd uptake into cells, possibly with or without the assistance of an unknown Cd transporter. Additionally, cytoplasmic HIPP19 may bind Cd²⁺, buffering free Cd²⁺ levels in the cytosol and potentially triggering feedback regulation of heavy metal-sensing pathways. In MIR408-OE plants, elevated miR408-5p represses HIPP19 protein accumulation, thereby disrupting Cd uptake. Purple clumps represent HIPP19 protein and blue barrel-shaped structures represent transporters. (TIFF) [file pbio.3003811.s010.tiff]

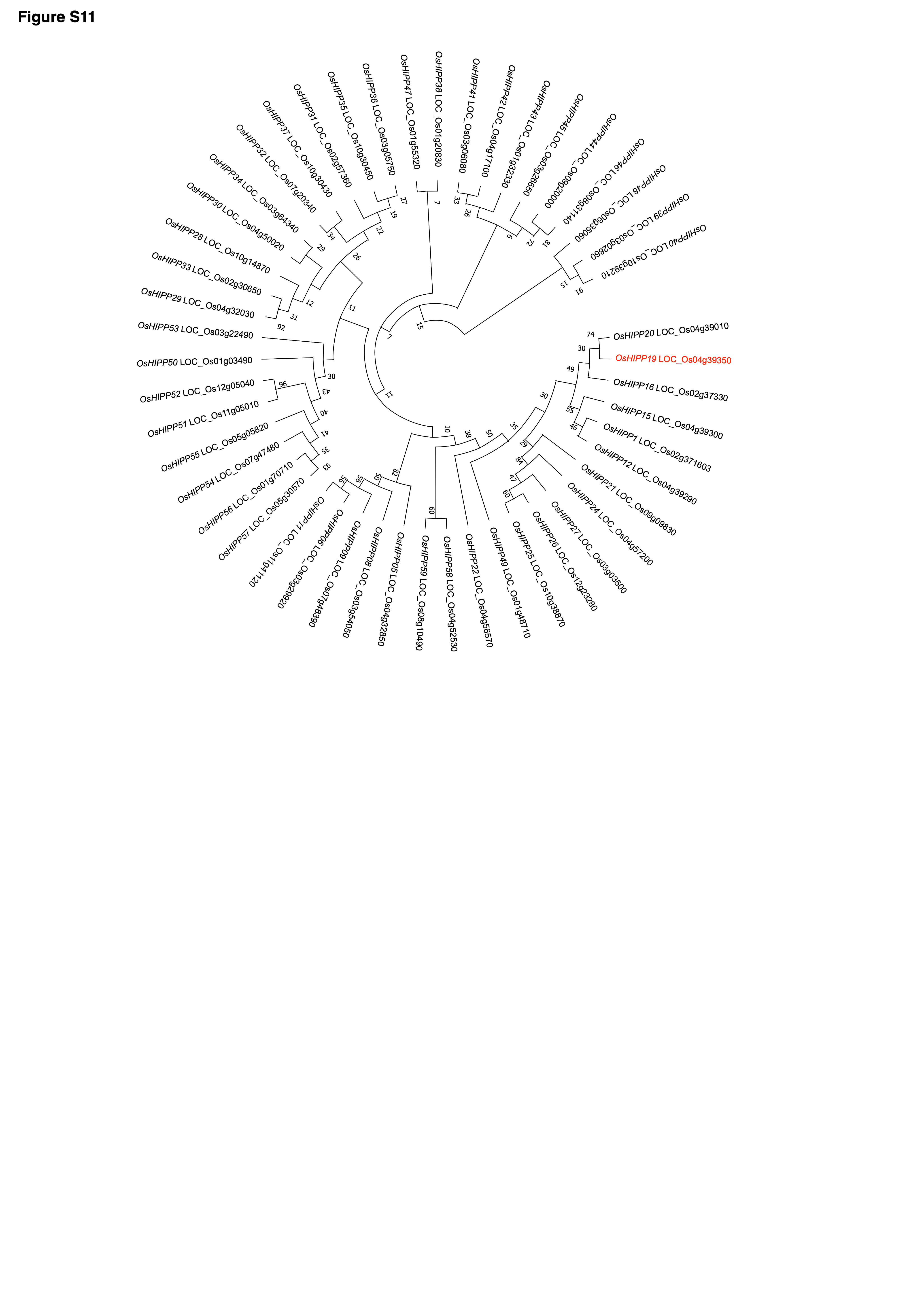

Supplement: S11 Fig — (TIFF) [file pbio.3003811.s011.tiff]

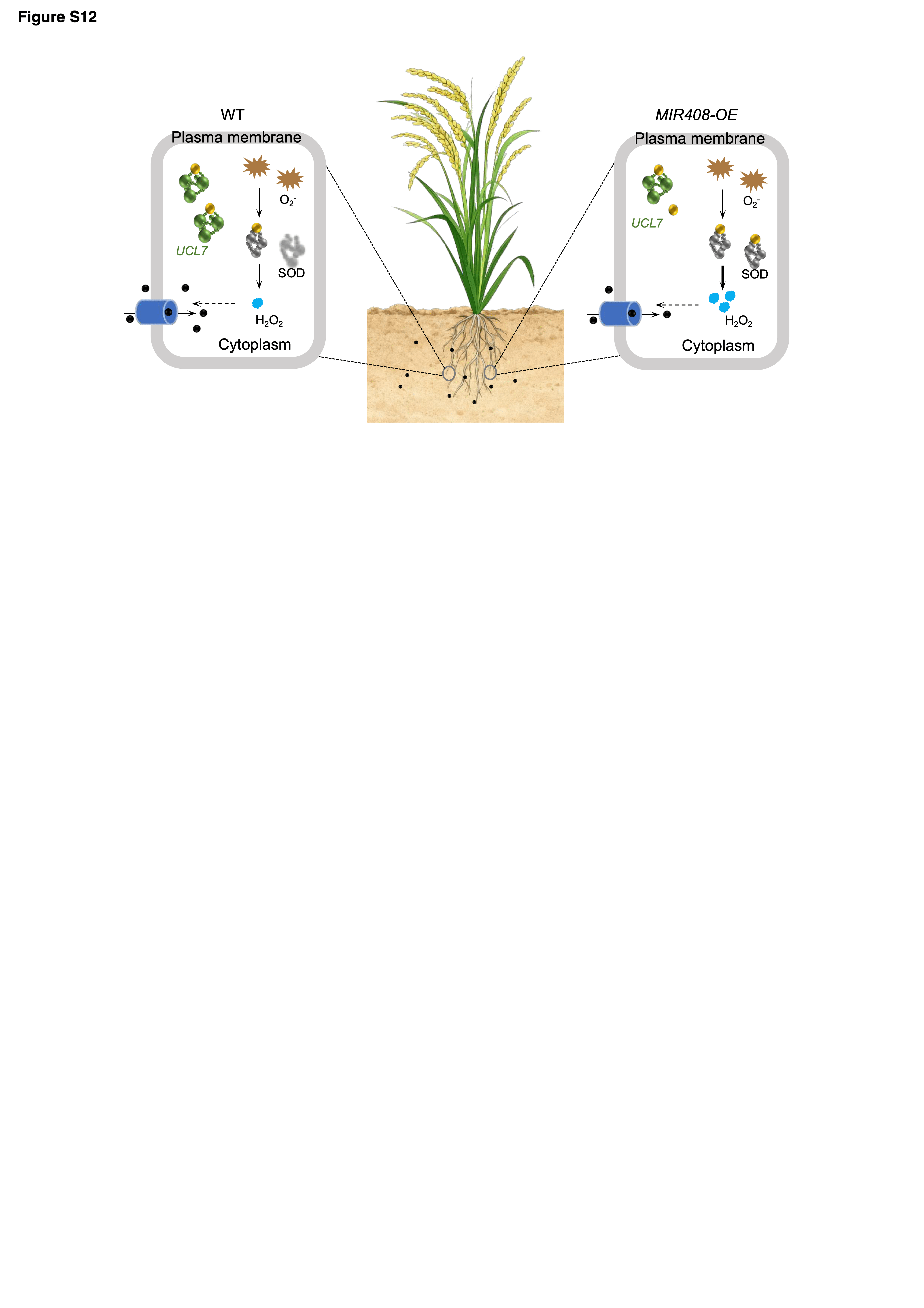

Supplement: S12 Fig — In WT plants, UCL7 may suppress SOD activity, resulting in low H2O2 production and consequently high Cd uptake. In MIR408-OE plants, elevated miR408-3p enhances cleavage of UCL7 transcripts, reducing the pool of UCL7 mRNA available for translation. As cytoplasmic UCL7 protein binds Cu²⁺, its depletion may increase intracellular copper availability, thereby boosting Cu/Zn SOD activity. These sequential events likely promote localized H2O2 production and the enhanced H2O2 accumulation in roots and shoots may modulate cation channel activity or alter heavy metal-related protein modifications, ultimately inhibiting Cd uptake. Green structures indicate UCL7 protein, gray structures denote SOD enzymes, black dots represent Cd²⁺ ions, and yellow dots represent Cu²⁺ ions. (TIFF) [file pbio.3003811.s012.tiff]

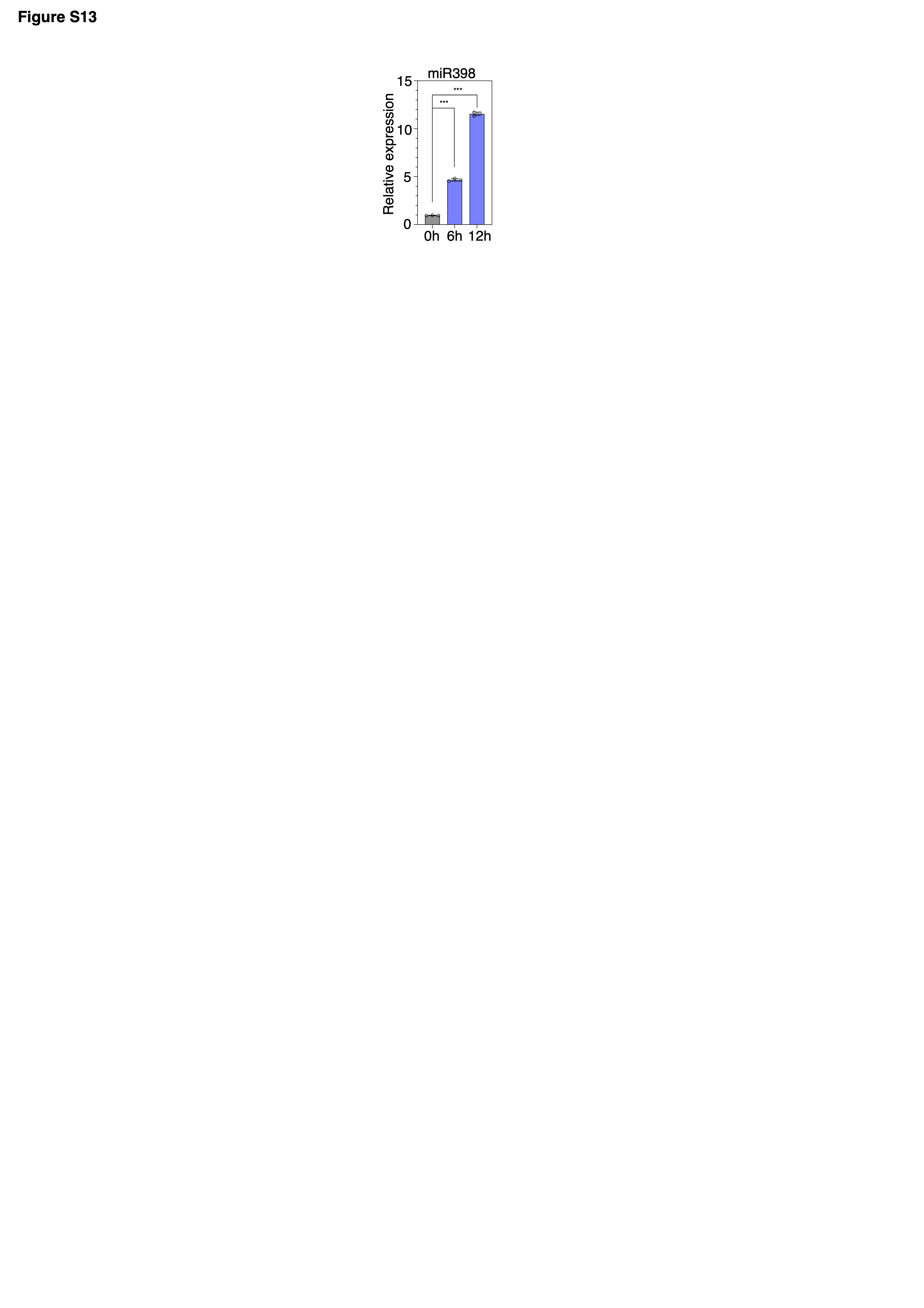

Supplement: S13 Fig — Student’s t test. ***P < 0.001. Values are means ± SD (n = 3 biological replicates). The data underlying this Figure can be found in S1 Data. (TIFF) [file pbio.3003811.s013.tiff]

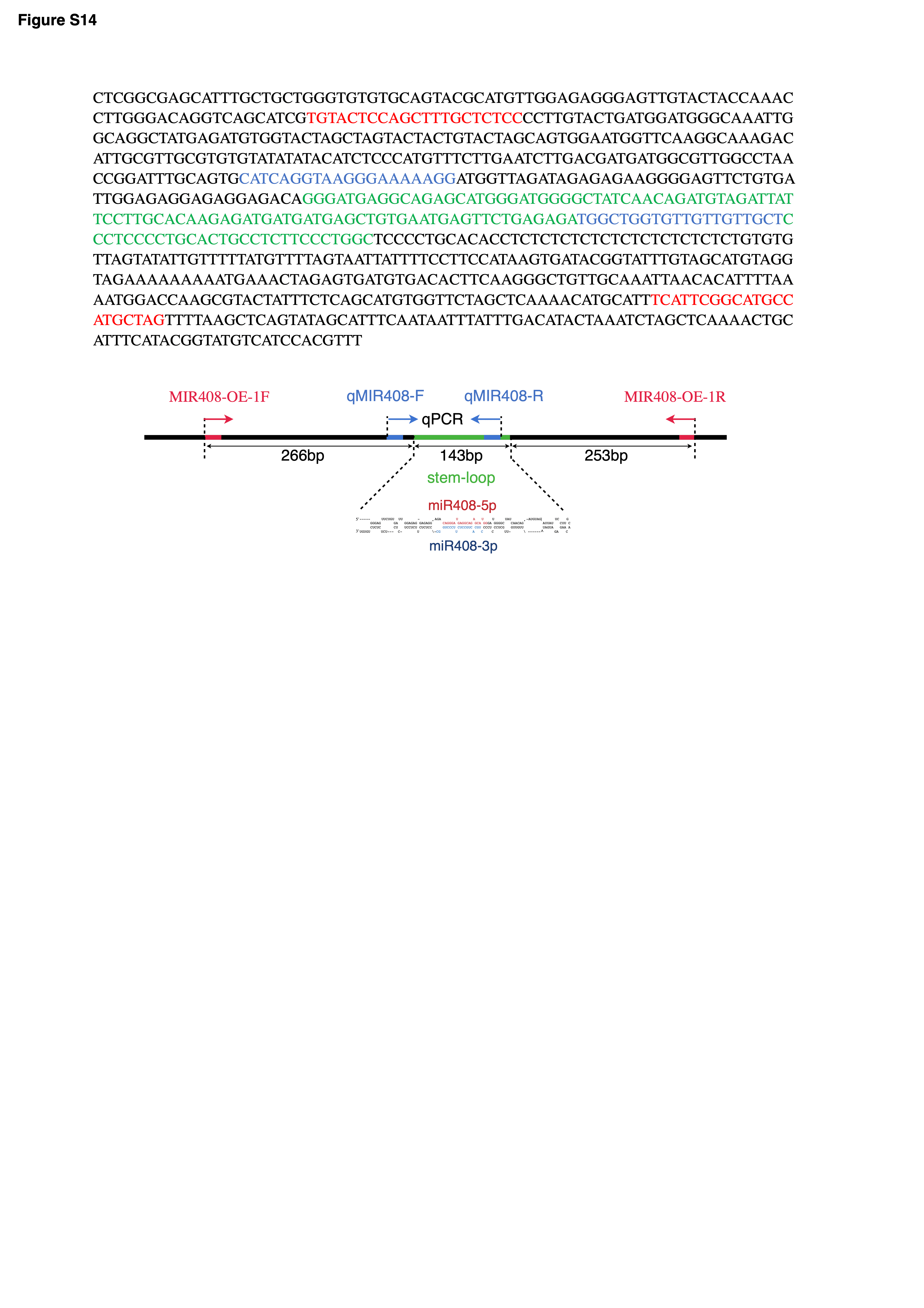

Supplement: S14 Fig — The upper sequences and lower lines highlighted in green represent the miR408 precursor sequence and its corresponding location, respectively. The upper sequences and lower lines marked in blue denote the primers used for detecting MIR408 expression and their respective positions, respectively. The upper sequences and lower lines marked in red represent the primers used for MIR408 overexpression and their respective positions, respectively. (TIFF) [file pbio.3003811.s014.tiff]

Fig 2G

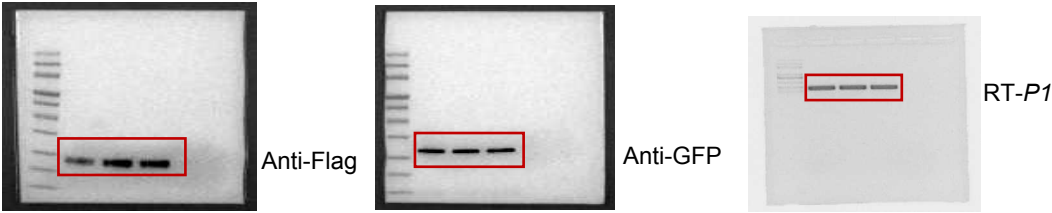

Fig 2H

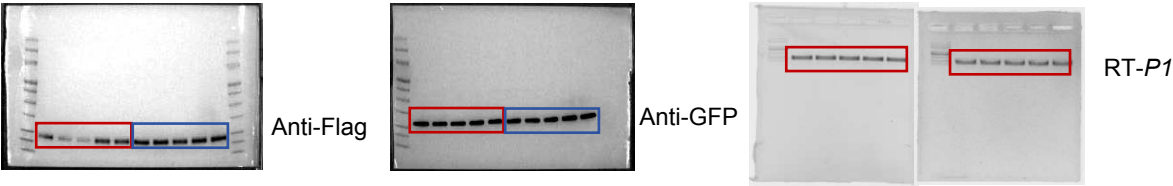

Fig 2I

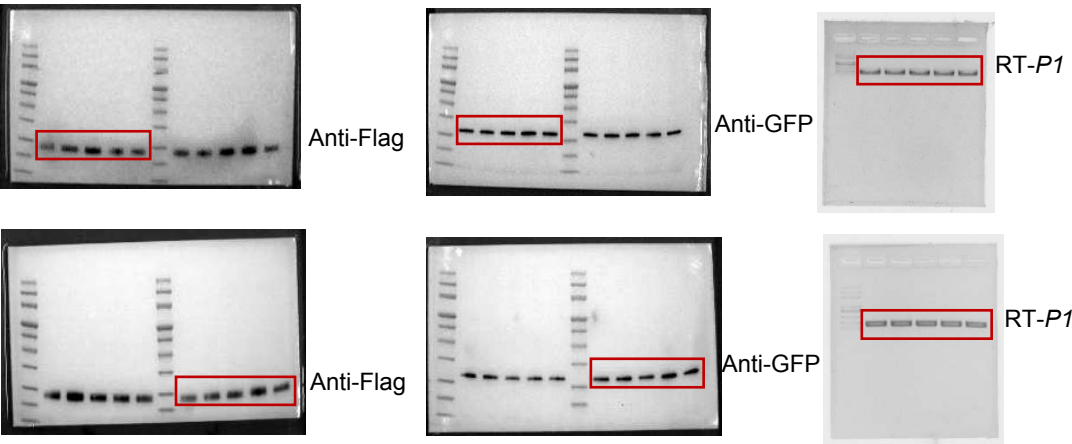

Fig 2K

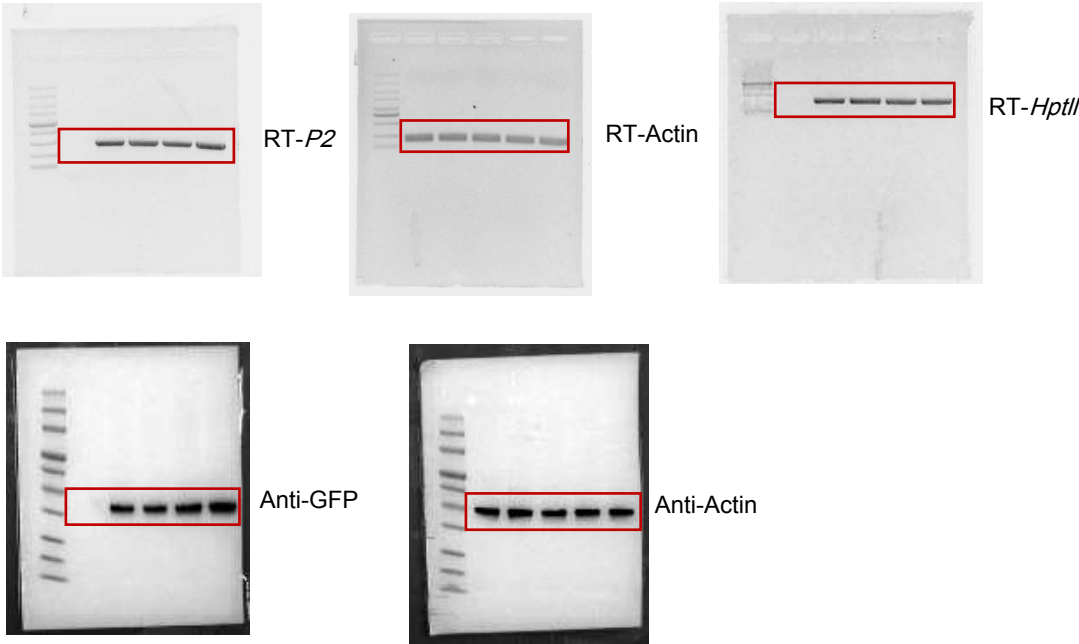

S2H Fig

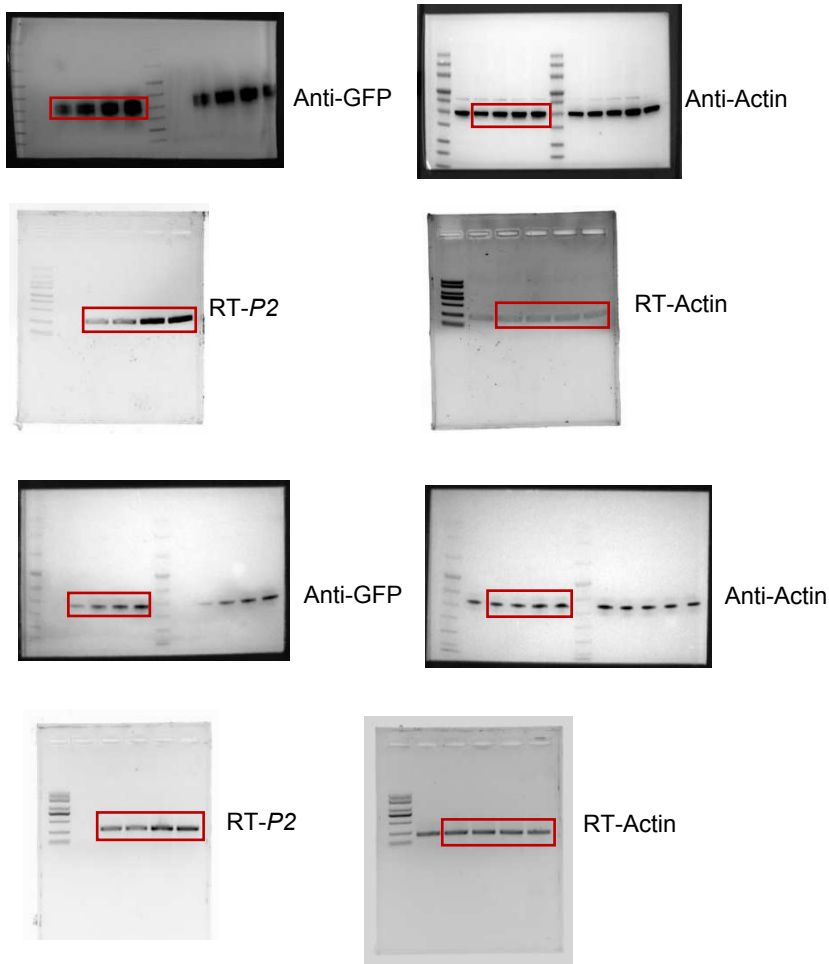

S2I Fig

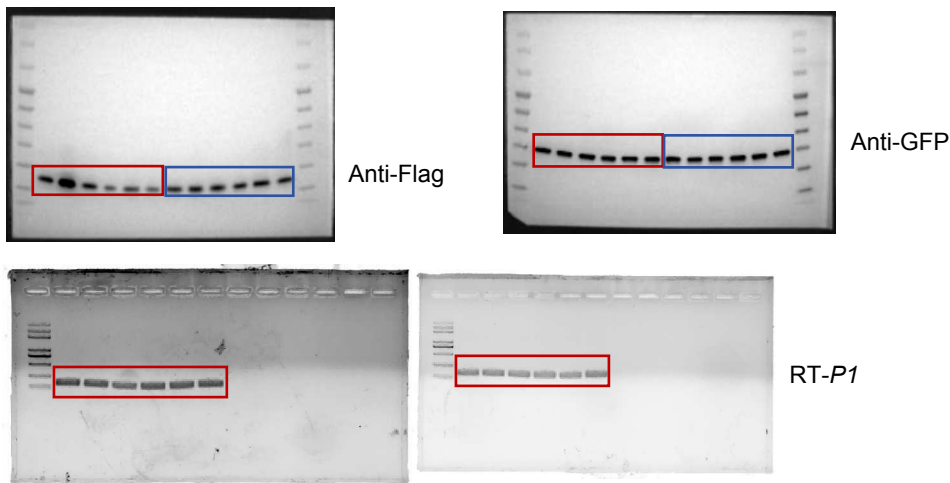

Supplement: S1 Raw Images — (PDF) [file pbio.3003811.s017.pdf]
